# Comparative efficacy and safety of mechanical circulatory support in ST-elevation myocardial infarction with cardiogenic shock: a network meta-analysis

**DOI:** 10.3389/fcvm.2026.1782887

**Published:** 2026-10-28

**Authors:** Boyu Guan, Xinsheng Li, Jiahua Zhang, Dongyuan Yu, Yujian Peng, Haichen Lv, Zhujing Hao

**Affiliations:** Department of Cardiology, The First Affiliated Hospital of Dalian Medical University, Dalian, China

**Keywords:** cardiogenic shock, critical cares, mechanical circulatory support, network meta-analysis, ST-segment elevation myocardial infarction

## Abstract

**Background:**

ST-segment elevation myocardial infarction (STEMI) with cardiogenic shock carries high mortality, yet optimal treatment remains uncertain in this distinct patient population. The aim of this study is to systematically evaluate and compare the efficacy of mechanical circulatory support (MCS) devices, vasoactive agents, and inotropic agents in the management of critical adult patients with STEMI complicating cardiogenic shock.

**Methods:**

A network meta-analysis (PROSPERO CRD420251002524) was conducted, with systematic searches of six databases (Embase, Web of Science, PubMed, and Cochrane, ClinicalTrials.gov, and the World Health Organization International Clinical Trials Registry Platform) from inception to March 2025 for randomized and observational studies. We also updated the search to September 2026 using the same search strategy.

**Results:**

Data from 22 studies (pooled *n* = 360,235) revealed a predominantly male (65%), older adult population (mean age 67 years), US-based (97.9%) population with hypertension, hyperlipidemia, diabetes or heart failure. Compared with intra-aortic balloon pump (IABP), based on observational evidence, Impella was associated with a significantly higher risk of short-term mortality (odds ratio [OR] 1.68, 95% confidence interval [CI] 1.49–1.89), bleeding (OR 1.62, 95% CI 1.49–1.75), stroke (OR 2.67, 95% CI 1.01–7.03), AKI (OR 1.55, 95% CI 1.10–2.18), and AKI requiring dialysis (OR 1.67, 95% CI 1.50–1.87). Compared with control, Impella significantly increased the risk of stroke (OR 4.37, 95% CI 3.74–5.11). Randomized controlled trials (RCTs) have also supported an increased bleeding risk with Impella compared with IABP or standard care.

**Conclusion:**

For STEMI patients with cardiogenic shock, IABP may be considered as a mechanical support option when hemodynamically feasible. By contrast, Impella has been associated with an unfavorable risk-benefit profile in this population in observational studies, which may be heavily influenced by patient selection and salvage use. Therefore, its application warrants careful consideration if escalation is necessary.

**Systematic Review Registration:**

https://www.crd.york.ac.uk/PROSPERO/view/CRD420251002524, PROSPERO CRD420251002524.

## Introduction

ST-segment elevation myocardial infarction (STEMI) complicated by cardiogenic shock represents a hemodynamically precarious state with prohibitively high short-term mortality (12-fold elevation in 30-day cardiovascular death events, *p* < 0.001) (1) compared to non-cardiogenic shock counterparts, representing a critical clinical scenario with high morbidity and mortality rates (2–6). Stabilization during the index hospitalization served as a critical determinant for achieving favorable longitudinal outcomes (1, 4). The role of mechanical circulatory support (MCS) devices such as intra-aortic balloon pump (IABP), Extracorporeal membrane oxygenation (ECMO), and Impella in managing this condition remains contentious, with their therapeutic benefits being a subject of considerable debate. The 2023 ESC and 2025 ACC/AHA/ACEP/NAEMSP/SCAI guidelines both recommend against routine IABP use in Acute Coronary Syndrome (ACS) patients with cardiogenic shock without mechanical complications, with the latter also advising against routine ECMO (Class III, Level B). (7, 8) In IABP-SHOCK II trial (9), IABP therapy provided no 30-day mortality benefit and demonstrated comparable secondary outcomes and safety profiles versus the no-IABP group in patients with acute myocardial infarction (AMI) and cardiogenic shock. A significant benefit was observed for 180-day survival in STEMI with cardiogenic shock patients and the composite cardiac endpoint in DanGer-SHOCK trial (10) with the microaxial flow pump over standard care, however the mortality advantage of the microaxial flow pump must be weighed against its safety concerns. In ECLS-SHOCK (11) and ECMO-CS (12) trials, routine early extracorporeal life support (ECLS) does not improve 30-day survival for patients with AMI related cardiogenic shock. Furthermore, ECLS-SHOCK demonstrated significantly higher risks of bleeding and vascular complications, while ECMO-CS did not find a statistically significant difference in these adverse events.

Findings of these randomized controlled trials (RCTs) are specific to cardiogenic shock complicating AMI (not exclusively STEMI,) and are frequently contradicted by real-world observational data and clinical practices (detailed in supplementary references). This divergence likely stems from fundamental differences in study design, patient selection, and management strategies. RCTs often enforce strict inclusion and exclusion criteria to ensure safety and homogeneity, which excludes the sickest, most complex patients typically seen in registry cohorts. Conversely, observational studies capture a broader spectrum of critical illness, including those with severe hemodynamic instability or multi-organ failure who are rarely randomized but represent a significant portion of real-world mortality. Furthermore, RCTs generally mandate rapid enrollment and protocol-driven interventions, whereas real-world delays in recognizing cardiogenic shock, transferring patients, or initiating support can lead to irreversible end-organ damage before therapy begins, negating potential benefits observed in idealized trial settings. This discrepancy warranted a thorough comparative assessment, particularly in the understudied context of critical STEMI precipitating cardiogenic shock, although AMI with cardiogenic shock has been more widely studied (13–18).

Current strategies for STEMI complicated by cardiogenic shock vary widely in their approach, with limited consensus on the optimal combination or standalone use of these interventions. Although existing studies have explored the application of MCS devices in STEMI patients with concomitant cardiogenic shock, they have often focused on individual devices (4). In a pairwise meta-analysis (19) of mixed evidence incorporating 2 RCTs and 5 observational studies, Impella demonstrated no significant advantage over IABP in reducing short-term mortality or stroke risk in patients with AMI and cardiogenic shock but was associated with a significantly higher risk of bleeding and limb complications. While such studies offer initial insights into the efficacy of certain devices, a comprehensive comparison across different devices, whether used individually or in combination, is lacking. An imperative exists to elucidate the optimal MCS device selection algorithm tailored to specific patient clinical phenotypes, thereby optimizing therapeutic outcomes while mitigating procedural risks.

The rationale for this review is to systematically evaluate and compare the efficacy of MCS devices, vasoactive agents, and inotropic agents in the management of critical adult patients with STEMI complicated by cardiogenic shock. By conducting a network meta-analysis (*N*MA), this review aims to provide a comprehensive comparison of these therapies, identifying the most effective strategies and potential synergies between them.

## Methods

### Protocol and registration

This investigation was prospectively catalogued in PROSPERO (CRD420251002524) (20), an international registry for systematic review protocols, and executed in adherence to “Meta-analysis Of Observational Studies in Epidemiology” (MOOSE) guidelines (21), thereby ensuring methodological integrity and transparency throughout the research process.

### Data sources and search strategies

A systematic search, conducted by two independent researchers (GB and LX), was performed across Embase, Web of Science, PubMed, and Cochrane, supplemented by scrutiny of ClinicalTrials.gov and the World Health Organization International Clinical Trials Registry Platform (ICTRP), spanning each database's inception through March 2025. We also updated the search to September 2026 using the same search strategy. Unpublished studies were also considered. The detailed search methodology is delineated in Supplementary Table S1.

### Eligibility criteria

Eligible studies comprised RCTs and cohort studies assessing MCS devices or pharmacological therapies in adults with STEMI and cardiogenic shock, with reported clinical outcomes. Trials were eligible if they focused solely on STEMI or reported data for a STEMI subgroup within broader inclusion criteria, such as a trial for all acute coronary syndromes. STEMI was defined as new or presumed new ST-elevation of ≥1 mm in ≥2 anatomically contiguous leads (measured at the J-point) in all leads other than V2-V3 and ≥2 mm in men ≥40 y, ≥2.5 mm in men <40 y, and ≥1.5 mm in women regardless of age in leads V2-V3 (7), while cardiogenic shock was characterized by a sustained (>30 min) systolic blood pressure (SBP) below 90 mmHg or the requirement of pharmacological support to maintain SBP above 90 mmHg due to cardiac dysfunction (22). Only English-language human studies were included. Trials were excluded if critical confounding factors were not adequately addressed or if patient distribution across treatment groups was significantly unbalanced. Citation tracking was performed on all relevant references. A rigorous specification of eligibility parameters (Population, Intervention, Comparison, Outcome, Study design, PICOS) was documented within the supplemental materials.

### Outcomes

The primary outcome was short-term mortality (30-day and in-hospital). Secondary outcomes included bleeding, stroke, 1-year mortality, acute kidney injury (AKI), and AKI requiring hemodialysis. Secondary outcome definitions followed study-specific criteria when available.

### Study selection and data extraction

Two independent reviewers (GB and LX) conducted title and abstract screening with subsequent full-text assessment of potentially eligible studies, with discrepancies resolved by a senior reviewer (LH). Ethical approval was not required for this study, as all participants had previously provided informed consent under prior approved protocols.

Data extraction was subsequently performed using a standardized form (Supplementary Table S2–S4) and yielded first author, publication year, sample size, study design, interventions, outcomes, participant demographics (such as age and sex), location (country, single or multi-center) and baseline profile (such as hypertension, infarct-related artery). Extracted data underwent independent verification by study investigators (GB and LX).

### Quality assessment

Two independent reviewers (GB and LX) conducted bias risk and evidence quality assessments, with inconsistencies settled by an additional reviewer (LH). Risk of bias of each individual study was assessed using the Newcastle-Ottawa Scale (*N*OS) (23) for cohort studies and the Cochrane RoB 2 tool (24) for randomized controlled trials. The GRADE rating (25) was utilized to rate evidence quality for direct and network meta-analysis outcomes. Thus, risk of bias and certainty of evidence were assessed using complementary study-level and outcome-level approaches.

### Statistical analysis

Discrete device-versus-device comparisons were analyzed through pairwise meta-analysis between all possible pairs. A sensitivity analysis was performed to identify any study that disproportionately affected the pooled effect estimate. Random-effect models were utilized. Publication bias was assessed using Egger's regression test, along with visual inspection of funnel, Labbe, and trim-and-fill plots. A network meta-analysis was conducted employing Markov Chain Monte Carlo (MCMC) computational methods using JAGS (Just Another Gibbs Sampler) for MCMC sampling. The model ran 4 chains of 25,000 iterations each. The first 5,000 iterations were discarded as burn-in (i.e., iterations 5001–25000 were retained), yielding 20,000 posterior samples per chain. Convergence was confirmed by inspecting Gelman-Rubin diagnostic plots, with all R-hat values below 1.05. Inconsistency was evaluated by analyzing differences in DIC (Deviance Information Criterion) values in both consistency and inconsistency model fits using node-splitting method, which separates direct and indirect evidence for each comparison (split node) to test whether the two sources of evidence disagree. For each split node, we calculated the difference between direct and indirect treatment effects along with 95% credible intervals (CrIs). In this NMA, the DIC values for the inconsistency and consistency models were 89.976 and 89.694. This difference is far less than 3, suggesting no evidence of inconsistency. The results of node-splitting also showed that all comparisons had a *P*-value greater than 0.05, with no significant inconsistency detected. Overall, these results do not suggest a violation of the transitivity assumption in this NMA. However, this does not establish transitivity. Surface Under the Cumulative Ranking Curve (SUCRA) transforms the mean rank into a value between 0% and 100%, with higher values indicating a greater probability of being among the more effective treatments. League tables and SUCRA curves were used to determine intervention rankings. All outcomes were dichotomous and we calculated odds ratios (ORs). Pairwise meta-analyses reported 95% confidence intervals (CIs), whereas Bayesian network meta-analyses reported 95% CrIs. A two-tailed *P* ≤ 0.05 indicated significance.

Data synthesis was performed in R (version 4.4.3) and RStudio with the following packages: “meta” and “metafor” for conventional meta-analysis, “gemtc” for network meta-analytical modeling, and “ggplot2” for data wrangling and visualization. The R code for meta-analysis is available in the supplement.

## Results

### Study selection

The study searching and selection process are detailed in the flow diagram (Figure 1). The final analysis synthesized evidence from 2 RCTs and 20 cohort studies, encompassing a pooled population of 360,235 patients.

**Figure 1:**
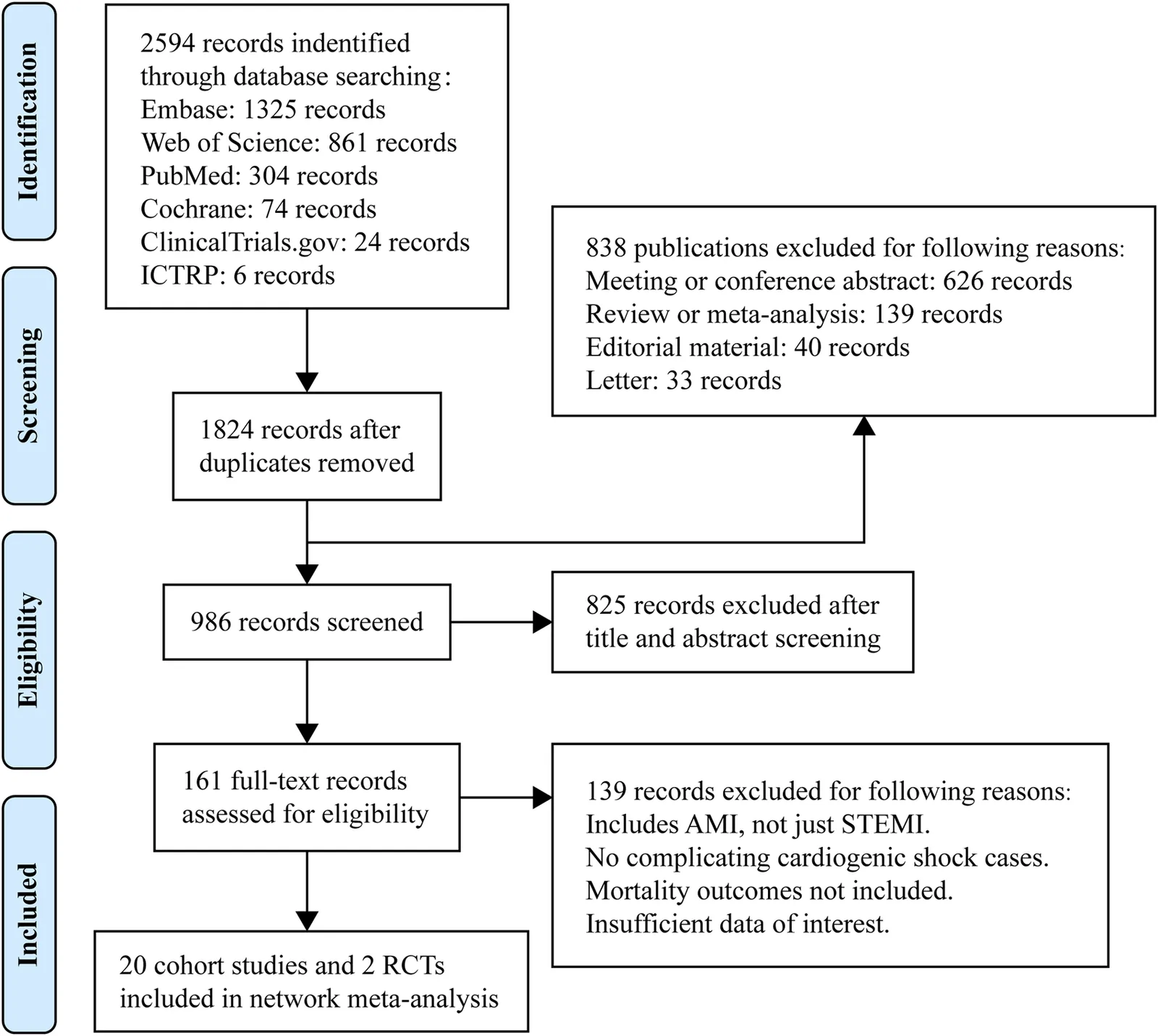
Flowchart of the searching and selection methodology.

### Study characteristics

Supplementary Table S2 presents the baseline characteristics of the studies included in the meta-analysis. The study population was overwhelmingly drawn from the United States (97.9%), exhibiting a mean age of 67 years with a male preponderance (65%). The demographic profiles for the trials are detailed in Supplementary Table S3 and Supplementary Table S4. Within the provided dataset, smoking (23.0–70.5%) persists as a pivotal yet modifiable risk factor. Hypertension (13.0–79.3%) emerges as the predominant comorbidity, with hyperlipidemia or dyslipidemia (17.2–53.0%), and diabetes (4.3–51.7%) also demonstrating notable prevalence. Anterior myocardial infarction (44.0–80.4%) constitutes the most frequent presentation of STEMI and ejection fraction (EF) is typically reduced. The left anterior descending artery (LAD, 28.0–87.0%) was the most commonly occluded vessel, followed by the right coronary artery (RCA, 10.3–70.5%) and the left circumflex (LCX, 4.3–62.6%), whereas left main coronary artery (LMCA, 0.8–31.9%) disease occurred less commonly. Multivessel obstruction (24.1–51.2%) demonstrates a higher incidence than solitary vessel compromise (12.5–59.8%). Angiographic success of percutaneous coronary intervention (PCI), assessed by post-procedural TIMI (thrombolysis in myocardial infarction) flow, was attained in most patients (56.0–99.9%). One trial implemented rate standardization to mitigate internal inconsistency (Supplementary Table S5).

### Risk of bias and evidence quality assessment

The majority of assessed domains manifested low risk of bias or acceptable concerns, as shown in Figure 2. In cohort studies, elevated risk of bias was primarily attributed to the inherent constraint of single-center research which may not adequately represent broader populations, or unresolved baseline characteristic disparities. In two RCTs, the insertion methods and visible differences among IABP, ECMO, and Impella devices pose significant challenges to effective blinding implementation. However, the primary endpoint is an objective measure (mortality), for which the impact of not using masking may be minimal. One study's comparability was deemed high-risk due to its concurrent assessment of three intervention arms and controls, resulting in evident intergroup discrepancies. An elaborated descriptive form is presented in the supplemental documentation.

**Figure 2:**
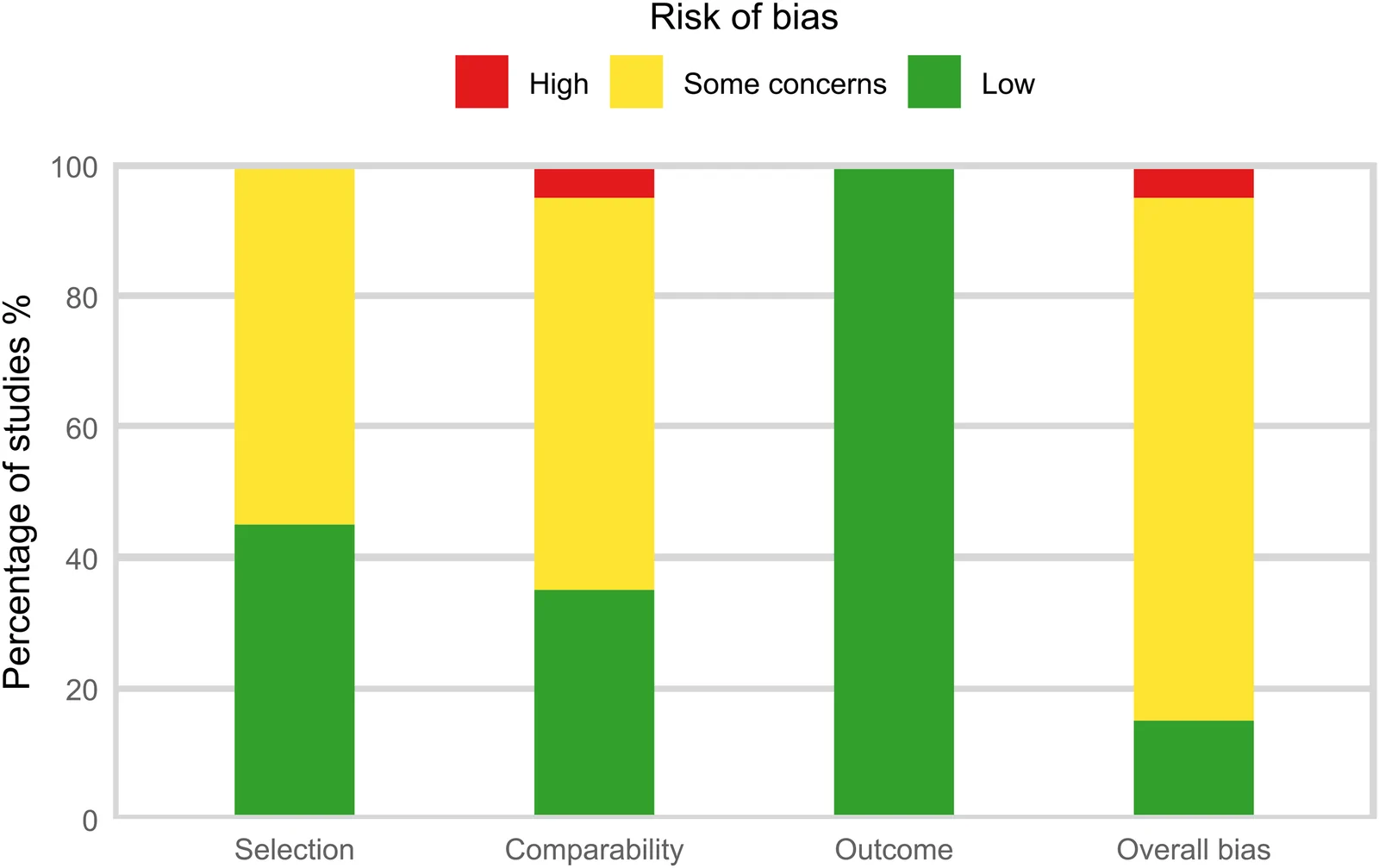
Overview of risk of bias across cohort studies.

GRADE criteria were applied to assess evidence quality across treatment groups for short-term mortality, bleeding, stroke, 1-year mortality, AKI, and AKI requiring hemodialysis (Supplementary Table S6). The aggregate evidence quality was limited, as observational studies intrinsically receive an initial low GRADE rating, further compounded by the paucity of available studies in this highly specialized clinical domain.

### Pairwise and network meta-analysis

The network plots appear in Supplementary Figure S1 for short-term mortality and Supplementary Figure S2 for secondary outcomes. Our synthesis identified three predominant therapeutic approaches (IABP, ECMO, Impella) with one unique study evaluating recombinant human B-type natriuretic peptide (rhBNP) therapy, whether administered individually or concomitantly. Among these, the IABP-Control comparison is the most rigorously investigated.

Results in Figure 3 exclusively reflect individual device performance, as combination therapy outcomes were seldomly reported in original research. Both NMA and traditional meta-analysis demonstrated no significant differences in short-term mortality, 1-year mortality, bleeding or stroke risk for IABP compared to control. ECMO was associated with a potential increase in short-term mortality compared to control, with effect estimates across both traditional (OR 2.42, 95% CI 2.25–2.61) and network (OR 1.32, 95% CrI 0.62–2.81) meta-analyses. Among the evaluated MCS options, Impella exhibited substantial heightened short-term mortality hazard versus IABP in pairwise meta (OR 1.51, 95% CI 1.10–2.06), along with significant higher hemorrhage (network meta, OR 2.55, 95% CrI 1.18–8.51) and stroke risks compared to control (pairwise meta, OR 3.44, 95% CI 1.56–7.56). Impella also confers a significant higher stroke risk than IABP (pairwise meta, OR 1.62, 95% CI 1.38–1.91). AKI risk was significantly higher in the Impella cohort compared with IABP recipients (pairwise meta, AKI: OR 1.55, 95% CI 1.10–2.18, and AKI requiring hemodialysis: OR 1.67, 95% CI 1.50–1.87).

**Figure 3:**
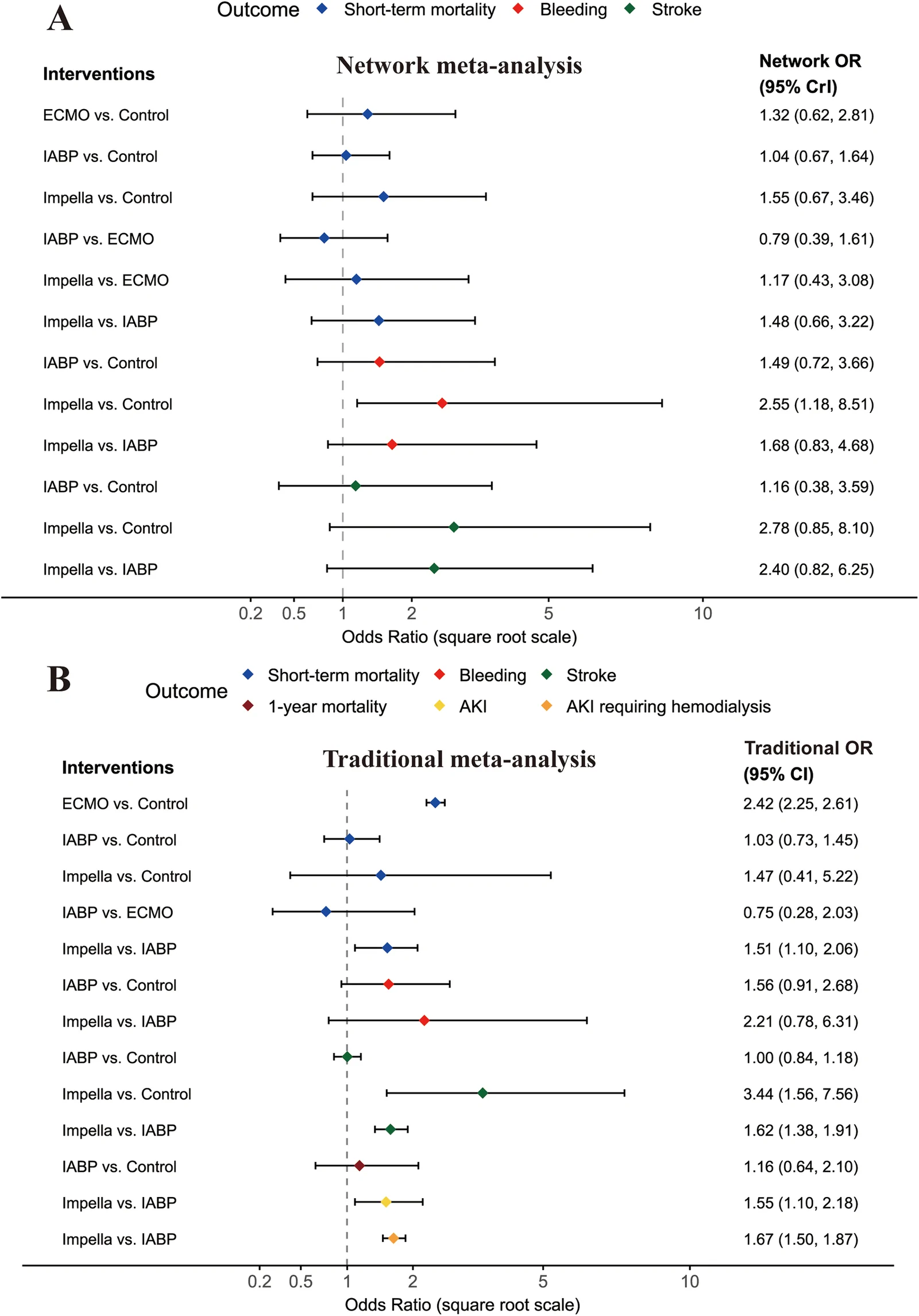
Pooled observational and RCT evidence. **(A)** Network meta-analysis, **(B)** Traditional meta-analysis.

Results derived from different sources of evidence (evidence drawn from RCTs, propensity-matched and overall observational studies, as well as adjusted observational estimates) were presented in Figure 4,5. RCT evidence demonstrated that, compared with control (standard care) and IABP, Impella significantly increased the risk of bleeding (compared with control: Relative Risk 2.06, 95% CI 1.15–3.66; compared with IABP: OR 5.50, 95% CI 1.03–29.45). Propensity-matched observational studies showed that IABP was associated with a significantly higher risk of short-term mortality compared with ECMO (OR 1.59, 95% CI 1.30–1.94), and that Impella significantly increased the risks of short-term mortality (OR 1.58, 95% CI 1.47–1.69), bleeding (OR 1.62, 95% CI 1.49–1.75), stroke (OR 1.63, 95% CI 1.38–1.91), AKI (OR 1.30, 95% CI 1.22–1.39), and AKI requiring dialysis (OR 1.56, 95% CI 1.36–1.78) compared with IABP. Adjusted observational evidence from a single study suggested that IABP significantly reduced the risk of short-term and one-year mortality compared with control, however, the other two studies did not find statistically significant differences. Table 1 summarized results from different evidence sources on the effects of MCS devices on clinical outcomes.

**Figure 4:**
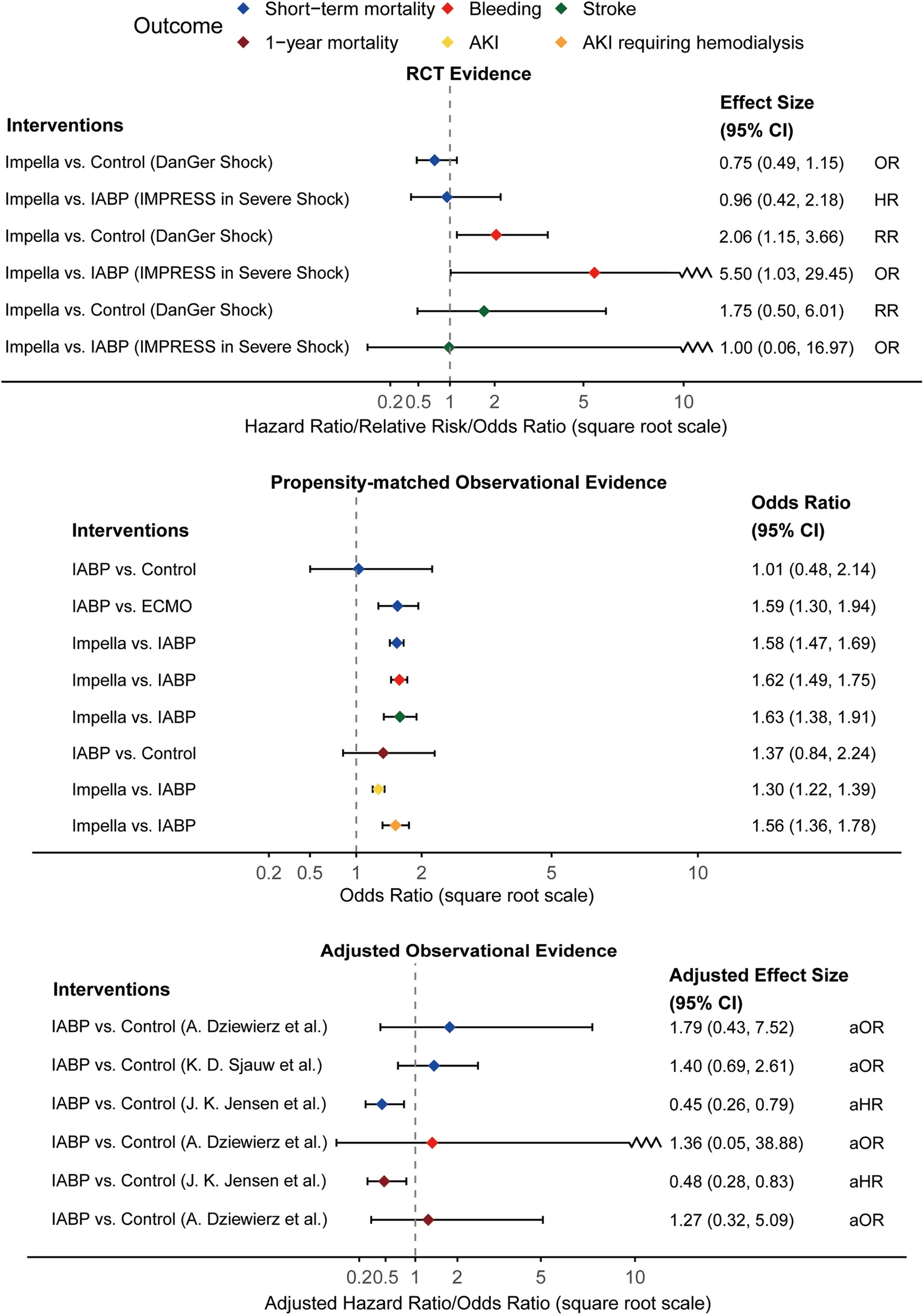
Pairwise meta-analysis of clinical outcomes (RCT evidence, propensity-matched and adjusted observational evidence).

**Figure 5:**
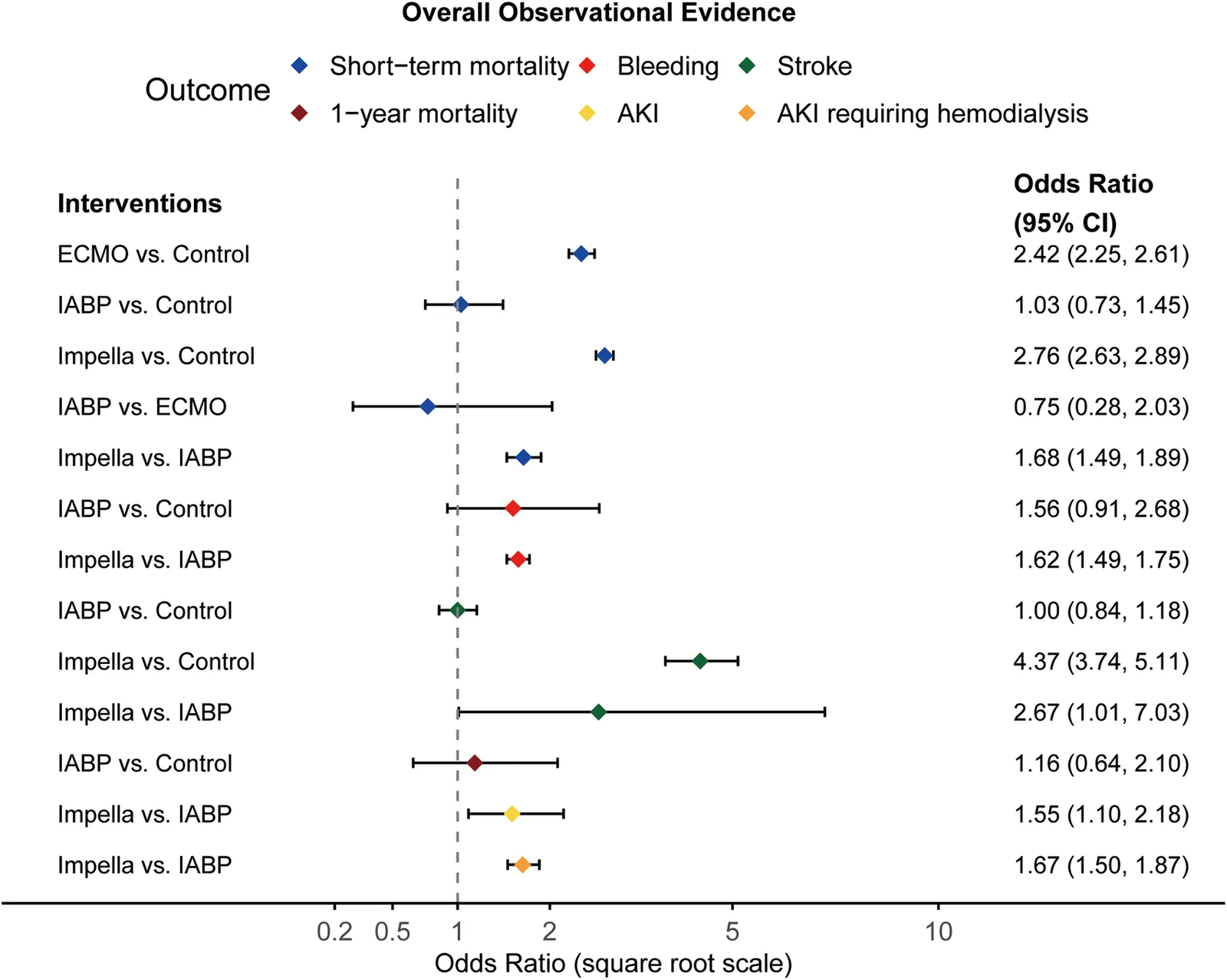
Pairwise meta-analysis of clinical outcomes (overall observational evidence).

**Table 1:** Comparison of clinical outcomes across different evidence sources.

	RCT and observational evidence	Overall observational	Propensity-matched observational	Adjusted observational	RCT evidence
(IABP vs. Control) Short-term mortality	NS	NS	NS	Control > IABP, NS, NS**	NR
(Impella vs. Control) Short-term mortality	NS	Impella > Control	NR	NR	NS
(ECMO vs. Control) Short-term mortality	ECMO > Control*	ECMO > Control	NR	NR	NR
(Impella vs. IABP) Short-term mortality	Impella > IABP*	Impella > IABP	Impella > IABP	NR	NS
(Impella vs. ECMO) Short-term mortality	NS	NR	NR	NR	NR
(IABP vs. ECMO) Short-term mortality	NS	NS	IABP > ECMO	NR	NR
(IABP vs. Control) Bleeding	NS	NS	NR	NS	NR
(Impella vs. Control) Bleeding	Impella > Control	NR	NR	NR	Impella > Control
(Impella vs. IABP) Bleeding	NS	Impella > IABP	Impella > IABP	NR	Impella > IABP
(IABP vs. Control) Stroke	NS	NS	NR	NR	NR
(Impella vs. Control) Stroke	Impella > Control*	Impella > Control	NR	NR	NS
(Impella vs. IABP) Stroke	Impella > IABP*	Impella > IABP	Impella > IABP	NR	NS
(IABP vs. Control) 1-year mortality	NS	NS	NS	Control > IABP, NS***	NR
(Impella vs. IABP) AKI	Impella > IABP	Impella > IABP	Impella > IABP	NR	NR
(Impella vs. IABP) AKI requiring dialysis	Impella > IABP	Impella > IABP	Impella > IABP	NR	NR^

AKI, acute kidney injury; ECMO, extracorporeal membrane oxygenation; IABP, intra-aortic balloon pump; NR, not reported (no data available); NS, not statistically significant; RCT, randomized controlled trial.

This table summarizes results from different evidence sources on the effects of mechanical circulatory support devices (including ECMO, Impella, IABP, and control) on clinical outcomes. “>“ indicates that the risk of the intervention on the left side of the symbol is higher than that on the right side.

We prioritized pairwise meta-analysis results over network meta-analysis estimates when discrepancies arose, as direct evidence is inherently more reliable than indirect evidence.

*Network meta-analysis showed no statistically significant difference. ** Two studies found no statistically significant difference. *** One study found no statistically significant difference.

^The DanGer SHOCK trial reported a higher rate of renal-replacement therapy in the Impella group than in the standard-care group (relative risk 1.98, 95% confidence interval 1.27–3.09).

Furthermore, an updated search through September 2026 identified a DanGer Shock sub-study showing Impella significantly increased bleeding events versus standard care (26.3% vs. 15.3%; *P* < 0.001).

Upon reviewing the league table (Table 2), seven therapeutic regimens including Control, ECMO, ECMO + Impella, IABP, IAB*P* + ECMO, Impella, and rhBNP + IABP were evaluated for short-term mortality outcomes. Complete league table results detailing all therapeutic comparisons are archived in Supplementary Table S7. Notably, ECMO + Impella emerged as the highest-risk strategy, with point estimates indicating 6-fold greater odds versus Control (OR 6.06, 95% CrI 1.03–35.28) and 4-fold versus ECMO or Impella alone. IABP + ECMO demonstrated the most favorable mortality outcome compared with other regimens, particularly when juxtaposed with ECMO + Impella (OR 0.04, 95% CrI 0–0.53). These findings were visually corroborated by rankograms and SUCRA values (Figure 6) and were further supported by a sensitivity analysis that excluded rhBNP.

**Table 2:** League table of network meta-analysis.

Intervention	Control	ECMO	ECMO + Impella	IABP	IABP + ECMO	Impella	rhBNP + IABP	Control	IABP	Impella
Control	Control	1.32 (0.62, 2.81)	6.06 (1.03, 35.28)	1.04 (0.67, 1.64)	0.25 (0.04, 1.68)	1.55 (0.67, 3.46)	0.61 (0.08, 4.64)	NA	NA	NA
ECMO	0.76 (0.36, 1.61)	ECMO	4.61 (0.72, 28.69)	0.79 (0.39, 1.61)	0.19 (0.02, 1.39)	1.17 (0.43, 3.08)	0.47 (0.06, 3.82)	NA	NA	NA
ECMO + Impella	0.17 (0.03, 0.97)	0.22 (0.03, 1.38)	ECMO + Impella	0.17 (0.03, 1)	0.04 (0, 0.53)	0.25 (0.05, 1.22)	0.1 (0.01, 1.44)	NA	NA	NA
IABP	0.96 (0.61, 1.49)	1.26 (0.62, 2.57)	5.81 (1, 33.53)	IABP	0.24 (0.04, 1.51)	1.48 (0.66, 3.22)	0.59 (0.08, 4.22)	NA	NA	NA
IABP + ECMO	4.01 (0.6, 28.27)	5.29 (0.72, 40.94)	24.36 (1.88, 318.56)	4.2 (0.66, 28.36)	IABP + ECMO	6.21 (0.83, 48.23)	2.48 (0.16, 37.86)	NA	NA	NA
Impella	0.65 (0.29, 1.48)	0.85 (0.32, 2.31)	3.92 (0.82, 18.81)	0.67 (0.31, 1.5)	0.16 (0.02, 1.21)	Impella	0.4 (0.05, 3.37)	NA	NA	NA
rhBNP + IABP	1.63 (0.22, 12.41)	2.14 (0.26, 17.86)	9.93 (0.69, 139.47)	1.69 (0.24, 12.45)	0.4 (0.03, 6.23)	2.52 (0.3, 20.91)	rhBNP + IABP	NA	NA	NA
Control	NA	NA	NA	NA	NA	NA	NA	Control	1.16 (0.38, 3.59) Stroke	2.78 (0.85, 8.1) Stroke
IABP	NA	NA	NA	NA	NA	NA	NA	0.67 (0.27, 1.39) Bleeding	IABP	2.4 (0.82, 6.25) Stroke
Impella	NA	NA	NA	NA	NA	NA	NA	0.39 (0.12, 0.84) Bleeding	0.6 (0.21, 1.2) Bleeding	Impella

Odds ratios (ORs) with 95% credible intervals are calculated. Values > 1.00 indicate higher odds of the outcome in the column group compared to the row group (column vs. row).

NA, not applicable.

**Figure 6:**
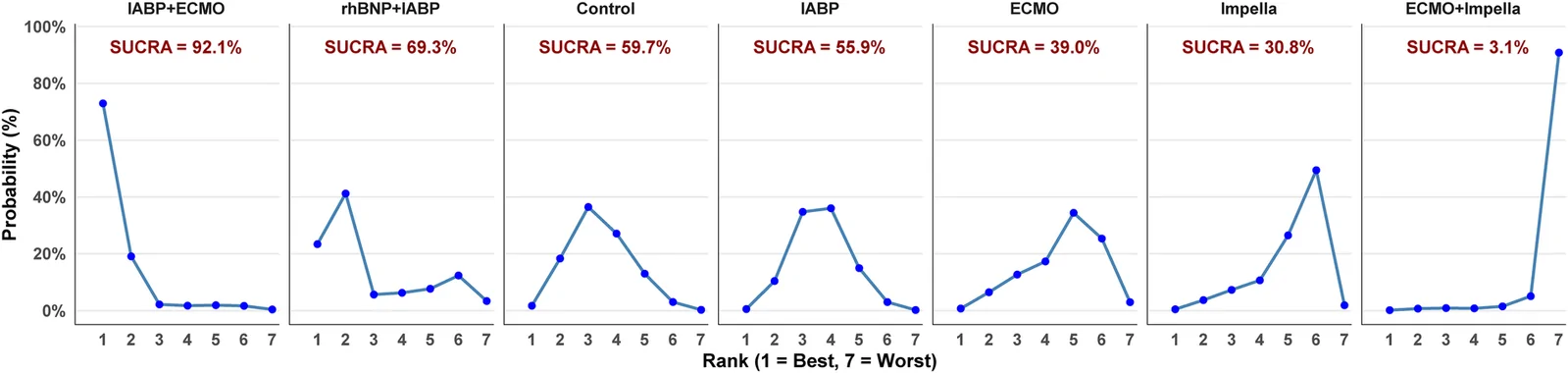
Rank probability plots and SUCRA values for short-term mortality endpoint.

Impella manifested substantially elevated hemorrhagic hazard surpassing conventional management, while displaying a risk aggravation relative to IABP. Parallel trends were observed for stroke endpoints.

### Sensitivity analysis and publication bias assessment

Detailed results of the pairwise meta-analysis, including forest, funnel, sensitivity, Labbe, and trim-and-fill plots, are available in the Supplementary Materials. To assess the robustness of the findings, a sensitivity analysis was performed. Studies exerting a disproportionate influence on the pooled effect estimate were identified and excluded. However, results for both the full and trimmed datasets are available in the Supplementary Materials. After excluding an influential study by Briasoulis et al., substantial heterogeneity was resolved, yielding I^2^ values of 0% for ECMO vs. control (short-term mortality), 13.5% for Impella vs. IABP (short-term mortality), and 0% for Impella vs. IABP (stroke). Similarly, exclusion of the study by Jensen et al. reduced heterogeneity in the IABP vs. control analysis for 1-year mortality to an I^2^ of 21.1%. A statistically significant difference was consistently observed in all four aforementioned subgroup analyses, with one exception: in the primary analysis including all eligible studies, ECMO was not significantly associated with short-term mortality compared with control (OR 1.65, 95% CI 0.83–3.29). After exclusion of the study by Briasoulis et al. in sensitivity analysis, heterogeneity was substantially reduced and the association became statistically significant (OR 2.42, 95% CI 2.25–2.61). No significant publication bias was detected by Egger's test across all available analyzed subgroups (*p* = 0.81, 0.94, 0.82, 0.20, 0.57, 0.60, 0.99, 0.56, 0.99), a finding corroborated by examination of funnel, Labbe, and trim-and-fill plots.

## Discussion

Cardiogenic shock complicating STEMI is a distinct clinical entity requiring customized hemodynamic support strategies, yet remains anomalously neglected in contemporary research. This investigation represents the inaugural systematic evaluation of MCS strategies exclusively in this critical population.

Through this systematic evaluation, pivotal findings emerge. The demonstrated hemorrhagic, cerebrovascular, and renal risks of Impella, as well as observed mortality trends, warrant careful clinical consideration when contemplating this device for individuals who had pre-existing or developing kidney or coagulation dysfunction. By contrast, IABP could be a reasonable alternative circulatory support option to Impella, if hemodynamics and other conditions permit. Preliminary data suggest possible efficacy for the IABP + ECMO combination in treating patients with STEMI-cardiogenic shock, but these findings require validation in larger, more robust studies.

Our findings are congruent with coeval (2023 and later) published studies. Au (26) and Nitta (27) discerned no significant advantage of Impella over IABP when used alongside ECMO, with similar mortality rates in cardiogenic shock patients. Similarly, Nishimoto (28) documented the superiority of ECMO + IABP, which not only reduced in-hospital mortality but also proved more economically efficient relative to ECMO + Impella, which had increased renal replacement therapy during hospitalization and more durable MCS implantations. Char (29) also reported the utility of IABP in cardiogenic shock, demonstrating that ECMO + IABP significantly enhanced 180-day survival compared to ECMO alone, whereas ECMO + Impella failed to yield a comparable benefit and showed a higher incidence of bleeding events. In addition, Movahed's (30) analysis favored IABP, associating it with lower mortality than Impella in patients with cardiogenic shock. Notwithstanding these observations, Unoki (31) reported that the ECPELLA configuration (ECMO + Impella) conferred a survival advantage at one year and abated myocardial injury in refractory cardiogenic shock due to AMI, suggesting a potential niche application in profoundly compromised patients.

From a hemodynamic perspective, Yu's (32) analysis showed that ECPELLA significantly increased total blood flow while ECMO + IABP maintained baseline flow rates. However, ECMO + IABP maintained optimal pulsatility and negligible shear stress elevation, in contrast to Impella's non-pulsatile flow profile characterized by turbulent hemodynamics and pathologically elevated shear forces, which could explain fewer malign vascular sequelae with IABP and higher hemolysis and bleeding rates with Impella. Gandhi's (33) meta-analysis echoed this, noting comparable mortality between Impella and IABP in VA-ECMO patients but higher bleeding and hemolysis risks with Impella. For HR-PCI and cardiogenic shock scenarios, Geppert (34) highlighted declining IABP use in view of the absence of mortality reduction or hemodynamic improvement in these clinical contexts, while Impella showed improved outcomes with accumulated operator proficiency and iterative device optimization. Finally, Acosta (35) emphasized staged MCS selection based on SCAI (the Society for Cardiovascular Angiography and Interventions) shock classification, which recommends IABP for early-stage shock and reserves advanced or combined MCS modalities for patients in more advanced stages (C-E) of hemodynamic deterioration, when combined IABP or Impella support with ECMO provides measurable clinical advantage in such high-stakes scenarios.

Several potential mechanisms may be involved in mediating the differing therapeutic efficacies of MCS modalities in STEMI complicated by cardiogenic shock, and were enumerated in the subsequent text and tabulated in Supplementary Tables S8 (26–35).

Impella device facilitates direct ventricular decompression through active blood aspiration from the left ventricle into the systemic circulation. While this mechanism offers more substantial hemodynamic support in theory, the clinical outcomes were less favorable with higher rates of adverse effects. These untoward effects may be attributed to a confluence of factors: the larger arterial access required for Impella insertion increases vascular complications such as bleeding; the device's mechanical action induces subclinical hemolysis, potentially exacerbating renal microvascular dysfunction; and the obligatory intensive anticoagulation regimen heightens bleeding propensity.

In contrast, IABP orchestrates its hemodynamic influence through diastolic augmentation and systolic unloading, thereby optimizing coronary perfusion gradients while simultaneously attenuating myocardial oxygen consumption. The relatively lower risks of bleeding and AKI associated with IABP may stem from its minimally invasive nature and reduced anticoagulation requirements compared to more aggressive circulatory support modalities.

ECMO provides complete cardiopulmonary support but collaterally imposes deleterious ventricular afterload augmentation (systolic loading), creating a pathophysiological paradox that may impair myocardial recovery. This mechanistic insight explains the device's underwhelming performance when employed in isolation. However, when synergistically combined with IABP, the two modalities demonstrate complementary physiological effects. ECMO sustains systemic perfusion while IABP unloads the left ventricle through precisely timed balloon deflation just before systole, reducing aortic pressure and ventricular afterload during ejection. This systolic unloading decreases myocardial oxygen demand while mitigating pulmonary congestion. Simultaneously, IABP enhances coronary blood flow through rapid balloon inflation at the onset of diastole, creating an augmented pressure wave that drives blood retrograde through the coronary arteries when vascular resistance is the lowest. Furthermore, by maintaining balloon inflation through early systole until just before aortic valve opening, IABP sustains diastolic augmentation while preparing for the next unloading cycle, thereby optimizing both coronary perfusion and ventricular ejection throughout the cardiac cycle. This therapeutic synergy simultaneously addresses the competing demands of circulatory support (ECMO) and ventricular unloading with coronary perfusion optimization (IABP), creating a hemodynamic environment conducive to myocardial salvage, which emerged as a potentially efficacious strategy in our analysis.

The complexity of these physiological considerations emphasizes the necessity of a thorough risk-benefit assessment when choosing circulatory support strategies, integrating both hemodynamic metrics and pathophysiological repercussions. In clinical practice, the decision to combine MCS devices, such as ECMO with IABP or Impella, is usually made in patients with severe or refractory cardiogenic shock where isolated support proves inadequate. ECMO provides full cardiopulmonary support but increases left ventricular afterload, which may precipitate pulmonary edema and hinder myocardial recovery. Therefore, an unloading device (IABP or Impella) is often added to mitigate this detrimental effect. IABP achieves ventricular unloading through diastolic augmentation and systolic unloading with minimal anticoagulation, making it suitable for hemodynamically stable patients with bleeding or renal concerns. In contrast, Impella offers more potent active decompression but carries higher risks of bleeding and renal problems. Thus, the ECMO + Impella configuration is generally reserved for patients with profound cardiogenic shock, whereas ECMO + IABP may be an option when a less-aggressive unloading strategy is desired.

The 2025 ACS Guideline classifies microaxial support as a reasonable option to reduce mortality in selected STEMI patients with severe or refractory cardiogenic shock, based on the results of the DanGer-SHOCK trial (7, 10). Beyond our focus on short-term mortality, which may not have allowed sufficient time for treatment effects to manifest, the apparent discrepancy between the 180-day survival benefit of Impella observed in the DanGer-SHOCK trial and the increased short-term mortality reported in this meta-analysis is likely attributable to fundamental differences in patient selection and device timing. DanGer-SHOCK strictly excluded comatose post-cardiac arrest patients, which is a cohort with inherently high mortality, and mandated Impella implantation prior to or during primary PCI, thereby intervening early in cardiogenic shock before irreversible end-organ damage occurred. In contrast, registry-based cohorts frequently capture real-world practice where Impella is deployed as a late bailout strategy post-PCI, often after prolonged hypoperfusion. This delayed implantation in critically ill patients introduces confounding, as the device is reserved for the sickest individuals. Consequently, this meta-analysis likely overestimates the perceived risk of Impella compared with other MCS devices or medications.

From a clinical intensivist's perspective, the consistently observed signal of excess harm associated with Impella must be interpreted in the context of indication bias inherent to real-world critical care practice. In the management of STEMI-complicated cardiogenic shock, Impella is not deployed arbitrarily but is typically reserved for patients with the most refractory hemodynamic collapse or as a salvage modality. Consequently, the Impella cohort in observational registries is enriched with the sickest patients who carry an intrinsically poorer prognosis. The device appears to confer excess risk because it is preferentially applied to those already at the greatest risk of adverse outcomes. Furthermore, the insertion of Impella itself carries procedural burdens, such as vascular access complications, that may compound the baseline vulnerability of this critically ill subgroup. Therefore, the unfavorable risk-benefit profile reported here should not be read as evidence that Impella is intrinsically deleterious.

The study underscores the importance of individualized treatment strategies based on patient-specific risk factors, such as renal function, bleeding risk, and hemodynamic stability. The analysis identifies a conspicuous absence of high-quality evidence on protracted clinical outcomes such as annual mortality rates, which should be addressed as a research priority moving forward. By addressing these gaps, the medical community can move toward more precise and effective management of STEMI complicated by cardiogenic shock.

Of note, RCTs remain the gold standard for identifying treatment effects under tightly controlled conditions, yet their internal validity often comes at the cost of external generalizability. In real-world settings, patients present with complex comorbidities, polypharmacy, and socioeconomic challenges that trial inclusion criteria rarely capture, leading to a disconnect known as the efficacy-effectiveness gap. This creates a challenge for clinical decision-making, as interventions that demonstrate robust effects in RCTs may yield unpredictable outcomes when deployed at scale. Addressing this disconnect requires a shift toward systematic integration of real-world data alongside experimental evidence to ensure that therapeutic benefits observed in idealized populations translate meaningfully to the heterogeneity of everyday practice.

### Limitations

Several limitations merit consideration in this study. Our strict focus on a homogenous STEMI cohort required the exclusion of some valuable mixed trials (9, 11, 12), as separate outcome data for this subgroup were unavailable. In the specific context of STEMI complicated by cardiogenic shock, transitivity of NMA deserves particular attention. Patients of this population often differ substantially with respect to baseline comorbidity, shock severity, revascularization strategies and so on. If such effect modifiers are unevenly distributed across treatment comparisons in the network, indirect estimates derived from NMA may be biased. The predominance of observational data (20 cohort studies versus 2 RCTs) may inherently limit their ability to support causal claims, as they cannot account for all potential sources of bias introduced by non-random group allocation. Replication in studies with randomized designs would strengthen confidence in our conclusions. However, a bias-free analysis is unattainable given the dearth of available studies. The clinical implementation of IABP + ECMO requires rigorous validation through large-scale, meticulously designed RCTs with stringent protocols. Notably, such patients present with critical, highly heterogeneous conditions and complex comorbidities, making truly unbiased RCTs exceptionally challenging to conduct, as they may be confounded by selection bias and ethical constraints. Broad confidence intervals for comparisons reflect uncertainty in the magnitude of treatment effects, likely due to small sample sizes in some subgroups or variability across studies. These underscores the need for larger, more homogeneous STEMI-specific datasets to improve precision. Publication bias was not a significant issue. With over 97% of included patients drawn from the U.S., we acknowledge the risk of overlap among studies utilizing shared administrative databases. Despite differing study designs, overlapping recruitment periods may result in double-counting, which could limit the generalizability of our findings beyond the U.S. healthcare context. In addition, although our search covered the full spectrum of vasoactive and inotropic agents, rhBNP was the sole agent with available evidence. Thus, this study is unable to comprehensively compare all such agents. Secondary endpoints with low event rates may lack statistical reliability. The worse outcomes which imply non-trivial iatrogenic harm in the ECPELLA group may likely be attributable to higher baseline morbidity, which may not be suitable for guiding clinical decisions regarding the disuse of such device strategy as it reflects patient selection rather than treatment effect. Variability in treatment protocols such as timing of MCS initiation may also affect comparability. The included studies did not report sufficient subgroup analyses for key baseline characteristics (such as renal impairment, multivessel disease), precluding formal subgroup meta-analyses. Future studies should prespecify these subgroups to enable stratified pooled analyses. Last but not least, despite no detected inconsistency and confirmed MCMC convergence in our NMA, transitivity is not guaranteed because of heterogeneity in shock severity, timing of support, cardiac arrest, and treatment selection. Therefore, as with any NMA, indirect evidence estimates and SUCRA rankings should be regarded as probabilistic guides rather than definitive evidence, and their interpretation requires careful consideration of both estimation precision and clinical significance. In sum, the evidence presented in this study demands circumspect analysis in light of the aforementioned limitations. In clinical practice, therapeutic decisions must often be individualized, accounting for individual patient needs and real-time clinical scenarios.

## Conclusion

While current evidence and guidelines predominantly address AMI, this study specifically focuses on STEMI patients with cardiogenic shock. In this network meta-analysis of predominantly observational data, IABP could be a reasonable mechanical support option compared with Impella when hemodynamically feasible. This finding is further supported by RCT and propensity-matched observational evidence. Observational analyses were generally associated with higher rates of short-term mortality, stroke, bleeding, and renal complications with Impella compared with IABP, although these estimates remain susceptible to confounding by indication and salvage use. Randomized evidence supports an increased bleeding risk, while evidence for mortality, stroke, and renal outcomes remains less certain. Notably, while the DanGer SHOCK trial showed improved 180-day survival with Impella versus standard care in STEMI-related cardiogenic shock (at the cost of higher complication rates), the IMPRESS in Severe Shock trial found no significant 30-day or 6-month survival benefit of Impella compared to IABP in this critical population. Caution is therefore warranted in its application. The IABP + ECMO combination emerged as a potential strategy. Physiologically, this approach combines the comprehensive cardiopulmonary support of ECMO with the left ventricular unloading capability of IABP, potentially reducing adverse events. However, its clinical adoption requires rigorous validation through high-quality RCTs due to extant evidence of limited strength.

Furthermore, due to issues such as confounding in predominantly observational data and potential patient overlap, although the GRADE rating was moderate in a few associations, the overall certainty of evidence is actually low. More studies are needed to further confirm these results, as there are too few RCTs targeting this specific critical STMEI and cardiogenic shock population. Overall, these findings should be interpreted as hypothesis-generating, underscoring the need for careful and individualized strategy selection and additional research to optimize circulatory support in such high-risk patients.

## Data Availability

The original contributions presented in the study are included in the article/Supplementary Material, further inquiries can be directed to the corresponding authors.
