# Supplementary material for: Comparative efficacy and safety of mechanical circulatory support in ST-elevation myocardial infarction with cardiogenic shock: a network meta-analysis
Source: Front Cardiovasc Med. 2026 Oct 28;13:1782887. doi: 10.3389/fcvm.2026.1782887 (PMC13752107; doi:10.3389/fcvm.2026.1782887)
Supplement: Supplementary file 1 [file Supplementaryfile1.docx]

**Supplemental Materials**

**Contents**

**Meta-analysis of Observational Studies in Epidemiology (MOOSE) Checklist**

**PICOS: Population, Intervention, Comparison, Outcome, Study design**

**Supplemental Table S1 Database Search Protocols**

**Supplemental Table S2 Baseline Characteristics of Studies Incorporated in the Meta-Analysis**

**Supplemental Table S3 Demographic Profiles of the Meta-Analyzed Study Populations**

**Supplemental Table S4 Angiographic Profiles of the Meta-Analyzed Study Populations**

**Supplemental Table S5 Rate Standardization in Study Petrovic, Milovan 2019**

**Supplemental Table S6 GRADE Evidence Assessment for Pooled Analyses**

**Supplemental Table S7 League Table of Network Meta-analysis**

**Supplemental Table S8 A Brief Overview Comparing MCS**

**Database-by-year Overlap Matrix**

**Supplemental Figure S1 Primary Outcome Comparative Evidence Network**

**Supplemental Figure S2 Secondary Outcomes Comparative Evidence Network**

**Newcastle - Ottawa Quality Assessment Scale for Cohort Studies**

**Version 2 of the Cochrane Tool for Assessing Risk of Bias in Randomized Trials (Rob 2) for Randomized Controlled Trials**

**R Script-Network Meta**

**R Script-Pairwise Meta**

**Gelman-Rubin Diagnostic Plot for Convergence Assessment**

**DIC Values for the Inconsistency and Consistency Models**

**Results of Node-splitting**

**Forest Plot, Funnel Plot, Sensitivity Analysis Plot, Labbe Plot, and Trimfill Plot**

**SUCRA Rank Plot (Sensitivity Analysis: rhBNP Excluded)**

**League Table of Treatment Comparisons (Sensitivity Analysis: rhBNP Excluded)**

**References of Included Studies**

# Meta-analysis of Observational Studies in Epidemiology (MOOSE) Checklist

| Item | Recommendation | Location reported |
| --- | --- | --- |
| Reporting of **background** should include | | |
| 1 | Problem definition | Intro. |
| 2 | Hypothesis statement | NR (unclear prior to investigation) |
| 3 | Description of study outcome(s) | Methods, Suppl. |
| 4 | Type of exposure or intervention used | Intro., Methods, Suppl. |
| 5 | Type of study designs used | Methods, Suppl. |
| 6 | Study population | Intro., Methods, Suppl. |
| Reporting of **search strategy** should include | | |
| 7 | Qualifications of searchers (eg, librarians and investigators) | Title page |
| 8 | Search strategy, including time period included in the synthesis and keywords | Methods, Fig. 1, Suppl. Table S1 |
| 9 | Effort to include all available studies, including contact with authors | Methods, Fig. 1, Suppl. Table S1 |
| 10 | Databases and registries searched | Methods, Fig. 1, Suppl. Table S1 |
| 11 | Search software used, name and version, including special features used (eg, explosion) | EndNote X9 |
| 12 | Use of hand searching (eg, reference lists of obtained articles) | Methods |
| 13 | List of citations located and those excluded, including justification | Methods, Fig. 1, Suppl. Table S1 |
| 14 | Method of addressing articles published in languages other than English | Methods, Suppl. |
| 15 | Method of handling abstracts and unpublished studies | Methods, Fig. 1, Suppl. |
| 16 | Description of any contact with authors | No reply |
| Reporting of **methods** should include | | |
| 17 | Description of relevance or appropriateness of studies assembled for assessing the hypothesis to be tested | Methods, Suppl. |
| 18 | Rationale for the selection and coding of data (eg, sound clinical principles or convenience) | Methods, Suppl. |
| 19 | Documentation of how data were classified and coded (eg, multiple raters, blinding and interrater reliability) | Methods |
| 20 | Assessment of confounding (eg, comparability of cases and controls in studies where appropriate) | Methods |
| 21 | Assessment of study quality, including blinding of quality assessors, stratification or regression on possible predictors of study results | Methods |
| 22 | Assessment of heterogeneity | Methods |
| 23 | Description of statistical methods (eg, complete description of fixed or random effects models, justification of whether the chosen models account for predictors of study results, dose response models, or cumulative meta analysis) in sufficient detail to be replicated | Methods |
| 24 | Provision of appropriate tables and graphics | Methods, all graphs |
| Reporting of **results** should include | | |
| 25 | Graphic summarizing individual study estimates and overall estimate | Graphs in Results, Suppl. Table S6-7 |
| 26 | Table giving descriptive information for each study included | Suppl. Table S2-4 |
| 27 | Results of sensitivity testing (eg, subgroup analysis) | Results, Suppl. Table S6, Suppl. |
| 28 | Indication of statistical uncertainty of findings | Results, Discussion, Suppl. Table S6, Suppl. |
| Reporting of **discussion** should include | | |
| 29 | Quantitative assessment of bias (eg, publication bias) | Results, Fig. 2, Suppl. Table S6, Suppl. |
| 30 | Justification for exclusion (eg, exclusion of non English language citations) | Methods, Fig. 1, Suppl. Table S1 |
| 31 | Assessment of quality of included studies | Results, Fig. 2, Suppl. Table S6, Suppl. |
| Reporting of **conclusions** should include | | |
| 32 | Consideration of alternative explanations for observed results | Discussion |
| 33 | Generalization of the conclusions (ie, appropriate for the data presented and within the domain of the literature review) | Discussion |
| 34 | Guidelines for future research | Discussion, Conclusion |
| 35 | Disclosure of funding source | Funding |
| Intro. = Introduction part; NR = not reported; Suppl. = supplemental materials (especially texts and the unlabeled) | | |

# PICOS: Population, Intervention, Comparison, Outcome, Study design

**Population**

*Included*

1. Adults aged ≥18.

2. Patients diagnosed with ST segment elevation myocardial infarction (STEMI), typically defined as new or presumed new ST-elevation of ≥1 mm in ≥2 anatomically contiguous leads (measured at the J-point) in all leads other than V2-V3 and ≥2 mm in men ≥40 y, ≥2.5 mm in men <40 y, and ≥1.5 mm in women regardless of age in leads V2-V3.

3. Patients diagnosed with cardiogenic shock, typically defined as a sustained (>30 minutes) systolic blood pressure (SBP) below 90 mmHg or the requirement of pharmacological support to maintain SBP above 90 mmHg due to cardiac dysfunction.

*Excluded*

1. Pediatric or adolescent populations.

2. Patients presenting with non-ST elevation acute coronary syndromes (ACS), including myocardial infarction without ST elevation (NSTEMI) or unstable angina. Studies with mixed populations that include conditions other than STEMI will also be excluded to maintain a focus on STEMI-specific management.

3. Patients without cardiogenic shock.

**Interventions or exposures**

*Included*

Treatment of cardiogenic shock, including mechanical circulatory support (MCS) devices (used alone or in combination) categorized as intra-aortic balloon pump (IABP), Impella, Tandem Heart Device (THD), extracorporeal membrane oxygenation (ECMO), Protek Duo, and CentriMag, alongside medical therapy (used alone or in combination) involving vasoactive and inotropic agents, given the overlapping mechanisms of these pharmacological interventions.

**Comparison or controls**

*Included*

Other MCS devices, medical therapy (used alone or in combination), placebo, or no treatment of interest in adult patients with STEMI complicated by cardiogenic shock.

**Outcomes**

Main outcomes: Short-term mortality (in-hospital or 30-day)

Additional outcomes: Including, but not restricted to, long-term mortality, major bleeding, recurrent myocardial infarction, stroke.

**Study design**

Both randomized and nonrandomized study types were included.

*Included*

Randomized controlled trials or cohort studies evaluating one or more interventions, used alone or in combination, including MCS devices (IABP, Impella, THD, ECMO, Protek Duo, and CentriMag) or medical therapies (vasoactive and inotropic agents), compared against each other, placebo, or no treatment in adult patients with STEMI complicated by cardiogenic shock. Only English-language human studies were included. Trials were excluded if critical confounding factors were not adequately addressed or if patient distribution across treatment groups was significantly unbalanced. Both published and unpublished studies were sought.

# Supplemental Table S1 Database Search Protocols

| **Search number** | **Query** | **Results** |
| --- | --- | --- |
| **Embase** | | |
| 1 | (('st'/exp OR st) AND ('elevation'/exp OR elevation) AND myocardial AND ('infarction'/exp OR infarction) OR 'stemi'/exp OR stemi OR (('st'/exp OR st) AND ('segment'/exp OR segment) AND ('elevation'/exp OR elevation) AND myocardial AND ('infarction'/exp OR infarction)) OR (('st'/exp OR st) AND elevated AND myocardial AND ('infarction'/exp OR infarction))) AND [humans]/lim AND [english]/lim | 85530 |
| 2 | shock, AND cardiogenic OR (cardiogenic AND shock) | 49090 |
| 3 | intra aortic' AND balloon AND pumping OR (intra AND aortic AND balloon AND pumping) OR (intraaortic AND balloon AND pumping) OR (balloon AND pumping, AND intraaortic) OR (pumping, AND intraaortic AND balloon) OR (pumping, AND 'intra aortic' AND balloon) OR (balloon AND pumping, AND 'intra aortic') OR (pumping, AND intra AND aortic AND balloon) | 1930 |
| 4 | extracorporeal AND membrane AND oxygenation OR (extracorporeal AND membrane AND oxygenations) OR (membrane AND oxygenation, AND extracorporeal) OR (ecmo AND extracorporeal AND membrane AND oxygenation) OR (extracorporeal AND life AND support) OR (extracorporeal AND life AND supports) OR (life AND support, AND extracorporeal) OR (ecmo AND treatment) OR (ecmo AND treatments) OR (treatment, AND ecmo) OR (ecls AND treatment) OR (ecls AND treatments) OR (treatment, AND ecls) OR (oxygenation, AND extracorporeal AND membrane) OR (venoarterial AND ecmo) OR (ecmo, AND venoarterial) OR (venoarterial AND ecmos) OR (venoarterial AND extracorporeal AND membrane AND oxygenation) OR (venovenous AND ecmo) OR (ecmo, AND venovenous) OR (venovenous AND ecmos) OR (venovenous AND extracorporeal AND membrane AND oxygenation) | 44611 |
| 5 | impella | 6070 |
| 6 | tandem AND heart | 8379 |
| 7 | protek AND duo | 182 |
| 8 | centrimag | 1257 |
| 9 | vasoconstrictor AND agents OR (agents, AND vasoconstrictor) OR (vasopressor AND agent) OR (agent, AND vasopressor) OR (vasoactive AND agonists) OR (agonists, AND vasoactive) OR (vasopressor AND agents) OR (agents, AND vasopressor) OR vasoconstrictors OR (vasoconstrictor AND drugs) OR (drugs, AND vasoconstrictor) OR (vasoconstrictor AND agent) OR (agent, AND vasoconstrictor) OR (vasoactive AND agonist) OR (agonist, AND vasoactive) OR vasoconstrictor OR (vasoconstrictor AND drug) OR (drug, AND vasoconstrictor) | 37623 |
| 10 | vasodilator AND agents OR (agents, AND vasodilator) OR (vasodilator AND drugs) OR (drugs, AND vasodilator) OR vasodilators OR vasorelaxants OR vasodilator OR (vasodilator AND agent) OR (agent, AND vasodilator) OR (vasodilator AND drug) OR (drug, AND vasodilator) OR vasorelaxant OR (vasoactive AND antagonists) OR (antagonists, AND vasoactive) | 70785 |
| 11 | cardiotonic AND agents OR (cardiotonic AND agent) OR (agent, AND cardiotonic) OR (myocardial AND stimulants) OR (cardiac AND stimulants) OR (cardioprotective AND agents) OR (cardiotonic AND drugs) OR cardiotonics OR (inotropic AND agents, AND positive AND cardiac) OR (cardiac AND stimulant) OR (stimulant, AND cardiac) OR (cardiotonic AND drug) OR (drug, AND cardiotonic) OR cardiotonic OR (cardioprotective AND agent) OR (agent, AND cardioprotective) OR (myocardial AND stimulant) OR (stimulant, AND myocardial) | 19110 |
| 12 | #3 OR #4 OR #5 OR #6 OR #7 OR #8 OR #9 OR #10 OR #11 | 176939 |
| 13 | #1 AND #2 AND #12 | 1325 |
| **Web of Science** | | |
| 1 | (((TS=(ST Elevation Myocardial Infarction)) OR TS=(STEMI)) OR TS=(ST Segment Elevation Myocardial Infarction)) OR TS=(ST Elevated Myocardial Infarction) | 63013 |
| 2 | (TS=(Shock, Cardiogenic)) OR TS=(Cardiogenic Shock) | 32234 |
| 3 | (((((((TS=(Intra-Aortic Balloon Pumping)) OR TS=(Intra Aortic Balloon Pumping)) OR TS=(Intraaortic Balloon Pumping)) OR TS=(Balloon Pumping, Intraaortic)) OR TS=(Pumping, Intraaortic Balloon)) OR TS=(Pumping, Intra-Aortic Balloon)) OR TS=(Balloon Pumping, Intra-Aortic)) OR TS=(Pumping, Intra Aortic Balloon) | 10457 |
| 4 | (((((((((((((((((((((TS=(Extracorporeal Membrane Oxygenation)) OR TS=(Extracorporeal Membrane Oxygenations)) OR TS=(Membrane Oxygenation, Extracorporeal)) OR TS=(ECMO Extracorporeal Membrane Oxygenation)) OR TS=(Extracorporeal Life Support)) OR TS=(Extracorporeal Life Supports)) OR TS=(Life Support, Extracorporeal)) OR TS=(ECMO Treatment)) OR TS=(ECMO Treatments)) OR TS=(Treatment, ECMO)) OR TS=(ECLS Treatment)) OR TS=(ECLS Treatments)) OR TS=(Treatment, ECLS)) OR TS=(Oxygenation, Extracorporeal Membrane)) OR TS=(Venoarterial ECMO)) OR TS=(ECMO, Venoarterial)) OR TS=(Venoarterial ECMOs)) OR TS=(Venoarterial Extracorporeal Membrane Oxygenation)) OR TS=(Venovenous ECMO)) OR TS=(ECMO, Venovenous)) OR TS=(Venovenous ECMOs)) OR TS=(Venovenous Extracorporeal Membrane Oxygenation) | 37684 |
| 5 | TS=(Impella) | 3443 |
| 6 | TS=(Tandem Heart) | 9866 |
| 7 | TS=(Protek Duo) | 58 |
| 8 | TS=(CentriMag) | 369 |
| 9 | (((((((((((((((((TS=(Vasoconstrictor Agents)) OR TS=(Agents, Vasoconstrictor)) OR TS=(Vasopressor Agent)) OR TS=(Agent, Vasopressor)) OR TS=(Vasoactive Agonists)) OR TS=(Agonists, Vasoactive)) OR TS=(Vasopressor Agents)) OR TS=(Agents, Vasopressor)) OR TS=(Vasoconstrictors)) OR TS=(Vasoconstrictor Drugs)) OR TS=(Drugs, Vasoconstrictor)) OR TS=(Vasoconstrictor Agent)) OR TS=(Agent, Vasoconstrictor)) OR TS=(Vasoactive Agonist)) OR TS=(Agonist, Vasoactive)) OR TS=(Vasoconstrictor)) OR TS=(Vasoconstrictor Drug)) OR TS=(Drug, Vasoconstrictor) | 49561 |
| 10 | (((((((((((((TS=(Vasodilator Agents)) OR TS=(Agents, Vasodilator)) OR TS=(Vasodilator Drugs)) OR TS=(Drugs, Vasodilator)) OR TS=(Vasodilators)) OR TS=(Vasorelaxants)) OR TS=(Vasodilator)) OR TS=(Vasodilator Agent)) OR TS=(Agent, Vasodilator)) OR TS=(Vasodilator Drug)) OR TS=(Drug, Vasodilator)) OR TS=(Vasorelaxant)) OR TS=(Vasoactive Antagonists)) OR TS=(Antagonists, Vasoactive) | 111875 |
| 11 | (((((((((((((((((TS=(Cardiotonic Agents)) OR TS=(Cardiotonic Agent)) OR TS=(Agent, Cardiotonic)) OR TS=(Myocardial Stimulants)) OR TS=(Cardiac Stimulants)) OR TS=(Cardioprotective Agents)) OR TS=(Cardiotonic Drugs)) OR TS=(Cardiotonics)) OR TS=(Inotropic Agents, Positive Cardiac)) OR TS=(Cardiac Stimulant)) OR TS=(Stimulant, Cardiac)) OR TS=(Cardiotonic Drug)) OR TS=(Drug, Cardiotonic)) OR TS=(Cardiotonic)) OR TS=(Cardioprotective Agent)) OR TS=(Agent, Cardioprotective)) OR TS=(Myocardial Stimulant)) OR TS=(Stimulant, Myocardial) | 56043 |
| 12 | #3 OR #4 OR #5 OR #6 OR #7 OR #8 OR #9 OR #10 OR #11 | 256760 |
| 13 | #1 AND #2 AND #12 and English (Languages) | 861 |
| **PubMed** | | |
| Search number | Query | Results |
| 3 | (((ST Elevation Myocardial Infarction[MeSH Terms]) OR (STEMI)) OR (ST Segment Elevation Myocardial Infarction)) OR (ST Elevated Myocardial Infarction) | 27521 |
| 4 | (Shock, Cardiogenic[MeSH Terms]) OR (Cardiogenic Shock) | 14399 |
| 5 | (((((((Intra-Aortic Balloon Pumping[MeSH Terms]) OR (Intra Aortic Balloon Pumping)) OR (Intraaortic Balloon Pumping)) OR (Balloon Pumping, Intraaortic)) OR (Pumping, Intraaortic Balloon)) OR (Pumping, Intra-Aortic Balloon)) OR (Balloon Pumping, Intra-Aortic)) OR (Pumping, Intra Aortic Balloon) | 3912 |
| 6 | (((((((((((((((((((((Extracorporeal Membrane Oxygenation[MeSH Terms]) OR (Extracorporeal Membrane Oxygenations)) OR (Membrane Oxygenation, Extracorporeal)) OR (ECMO Extracorporeal Membrane Oxygenation)) OR (Extracorporeal Life Support)) OR (Extracorporeal Life Supports)) OR (Life Support, Extracorporeal)) OR (ECMO Treatment)) OR (ECMO Treatments)) OR (Treatment, ECMO)) OR (ECLS Treatment)) OR (ECLS Treatments)) OR (Treatment, ECLS)) OR (Oxygenation, Extracorporeal Membrane)) OR (Venoarterial ECMO)) OR (ECMO, Venoarterial)) OR (Venoarterial ECMOs)) OR (Venoarterial Extracorporeal Membrane Oxygenation)) OR (Venovenous ECMO)) OR (ECMO, Venovenous)) OR (Venovenous ECMOs)) OR (Venovenous Extracorporeal Membrane Oxygenation) | 20789 |
| 7 | Impella | 1378 |
| 8 | Tandem Heart | 2509 |
| 9 | Protek Duo | 20 |
| 10 | CentriMag | 183 |
| 11 | (((((((((((((((((Vasoconstrictor Agents[MeSH Terms]) OR (Agents, Vasoconstrictor)) OR (Vasopressor Agent)) OR (Agent, Vasopressor)) OR (Vasoactive Agonists)) OR (Agonists, Vasoactive)) OR (Vasopressor Agents)) OR (Agents, Vasopressor)) OR (Vasoconstrictors)) OR (Vasoconstrictor Drugs)) OR (Drugs, Vasoconstrictor)) OR (Vasoconstrictor Agent)) OR (Agent, Vasoconstrictor)) OR (Vasoactive Agonist)) OR (Agonist, Vasoactive)) OR (Vasoconstrictor)) OR (Vasoconstrictor Drug)) OR (Drug, Vasoconstrictor) | 109072 |
| 12 | (((((((((((((Vasodilator Agents[MeSH Terms]) OR (Agents, Vasodilator)) OR (Vasodilator Drugs)) OR (Drugs, Vasodilator)) OR (Vasodilators)) OR (Vasorelaxants)) OR (Vasodilator)) OR (Vasodilator Agent)) OR (Agent, Vasodilator)) OR (Vasodilator Drug)) OR (Drug, Vasodilator)) OR (Vasorelaxant)) OR (Vasoactive Antagonists)) OR (Antagonists, Vasoactive) | 212813 |
| 13 | (((((((((((((((((Cardiotonic Agents[MeSH Terms]) OR (Cardiotonic Agent)) OR (Agent, Cardiotonic)) OR (Myocardial Stimulants)) OR (Cardiac Stimulants)) OR (Cardioprotective Agents)) OR (Cardiotonic Drugs)) OR (Cardiotonics)) OR (Inotropic Agents, Positive Cardiac)) OR (Cardiac Stimulant)) OR (Stimulant, Cardiac)) OR (Cardiotonic Drug)) OR (Drug, Cardiotonic)) OR (Cardiotonic)) OR (Cardioprotective Agent)) OR (Agent, Cardioprotective)) OR (Myocardial Stimulant)) OR (Stimulant, Myocardial) | 78725 |
| 14 | #5 OR #6 OR #7 OR #8 OR #9 OR #10 OR #11 OR #12 OR #13 | 386573 |
| 15 | #3 AND #4 AND #14 | 304 |
| **Cochrane** | | |
| 1 | (ST Elevation Myocardial Infarction):ti,ab,kw OR (ST Segment Elevation Myocardial Infarction):ti,ab,kw OR (ST Elevated Myocardial Infarction):ti,ab,kw OR (STEMI):ti,ab,kw (Word variations have been searched) | 8199 |
| 2 | (Shock, Cardiogenic):ti,ab,kw OR (Cardiogenic Shock):ti,ab,kw | 1748 |
| 3 | (Intra-Aortic Balloon Pumping):ti,ab,kw OR (Pumping, Intra-Aortic Balloon):ti,ab,kw OR (Intraaortic Balloon Pumping):ti,ab,kw OR (Pumping, Intraaortic Balloon):ti,ab,kw OR (Balloon Pumping, Intraaortic):ti,ab,kw | 264 |
| 4 | (Intra Aortic Balloon Pumping):ti,ab,kw OR (Balloon Pumping, Intra-Aortic):ti,ab,kw OR (Pumping, Intra Aortic Balloon):ti,ab,kw | 250 |
| 5 | #3 OR #4 | 264 |
| 6 | (Extracorporeal Membrane Oxygenation):ti,ab,kw OR (Venovenous ECMO):ti,ab,kw OR (Venovenous Extracorporeal Membrane Oxygenation):ti,ab,kw OR (ECMO, Venovenous):ti,ab,kw OR (Venovenous ECMOs):ti,ab,kw | 1047 |
| 7 | (ECMO, Venoarterial):ti,ab,kw OR (Venoarterial Extracorporeal Membrane Oxygenation):ti,ab,kw OR (Venoarterial ECMOs):ti,ab,kw OR (Venoarterial ECMO):ti,ab,kw OR (Membrane Oxygenation, Extracorporeal):ti,ab,kw | 1049 |
| 8 | (ECLS Treatment):ti,ab,kw OR (ECMO Treatment):ti,ab,kw OR (ECMO Treatments):ti,ab,kw OR (Extracorporeal Life Support):ti,ab,kw OR (Treatment, ECMO):ti,ab,kw | 742 |
| 9 | (Life Support, Extracorporeal):ti,ab,kw OR (ECMO Extracorporeal Membrane Oxygenation):ti,ab,kw OR (Extracorporeal Membrane Oxygenations):ti,ab,kw OR (Oxygenation, Extracorporeal Membrane):ti,ab,kw OR (ECLS Treatments):ti,ab,kw | 1133 |
| 10 | (Extracorporeal Life Supports):ti,ab,kw OR (Treatment, ECLS):ti,ab,kw | 54 |
| 11 | #6 OR #7 OR #8 OR #9 OR #10 | 1393 |
| 12 | (Impella):ti,ab,kw | 177 |
| 13 | (Tandem Heart):ti,ab,kw | 323 |
| 14 | (Protek Duo):ti,ab,kw | 0 |
| 15 | (CentriMag):ti,ab,kw | 2 |
| 16 | (Vasoconstrictor Agents):ti,ab,kw OR (Drug, Vasoconstrictor):ti,ab,kw OR (Vasoconstrictor Agent):ti,ab,kw OR (Vasopressor Agent):ti,ab,kw OR (Agent, Vasopressor):ti,ab,kw | 2790 |
| 17 | (Vasoconstrictor Drugs):ti,ab,kw OR (Drugs, Vasoconstrictor):ti,ab,kw OR (Vasoactive Agonist):ti,ab,kw OR (Vasoconstrictor Drug):ti,ab,kw OR (Agents, Vasopressor):ti,ab,kw | 2651 |
| 18 | (Agonists, Vasoactive):ti,ab,kw OR (Vasopressor Agents):ti,ab,kw OR (Agent, Vasoconstrictor):ti,ab,kw OR (Agents, Vasoconstrictor):ti,ab,kw OR (Agonist, Vasoactive):ti,ab,kw | 2550 |
| 19 | (Vasoactive Agonists):ti,ab,kw OR (Vasoconstrictors):ti,ab,kw OR (Vasoconstrictor):ti,ab,kw | 3247 |
| 20 | #16 OR #17 OR #18 OR #19 | 3619 |
| 21 | (Vasodilator Agents):ti,ab,kw OR (Vasodilator Drugs):ti,ab,kw OR (Vasodilator Drug):ti,ab,kw OR (Agent, Vasodilator):ti,ab,kw OR (Drugs, Vasodilator):ti,ab,kw | 6165 |
| 22 | (Drug, Vasodilator):ti,ab,kw OR (Vasodilator Agent):ti,ab,kw OR (Agents, Vasodilator):ti,ab,kw OR (Vasoactive Antagonists):ti,ab,kw OR (Antagonists, Vasoactive):ti,ab,kw | 6209 |
| 23 | (Vasodilators):ti,ab,kw OR (Vasorelaxants):ti,ab,kw OR (Vasorelaxant):ti,ab,kw OR (Vasodilator):ti,ab,kw | 7201 |
| 24 | #21 OR #22 OR #23 | 7301 |
| 25 | (Cardiotonic Agents):ti,ab,kw OR (Inotropic Agents, Positive Cardiac):ti,ab,kw OR (Cardioprotective Agent):ti,ab,kw OR (Cardiotonic Agent):ti,ab,kw OR (Cardioprotective Agents):ti,ab,kw | 1947 |
| 26 | (Agent, Cardiotonic):ti,ab,kw OR (Drug, Cardiotonic):ti,ab,kw OR (Myocardial Stimulant):ti,ab,kw OR (Stimulant, Cardiac):ti,ab,kw OR (Myocardial Stimulants):ti,ab,kw | 1380 |
| 27 | (Agent, Cardioprotective):ti,ab,kw OR (Cardiotonic Drug):ti,ab,kw OR (Stimulant, Myocardial):ti,ab,kw OR (Cardiotonic Drugs):ti,ab,kw OR (Cardiac Stimulants):ti,ab,kw | 1578 |
| 28 | (Cardiac Stimulant):ti,ab,kw OR (Cardiotonics):ti,ab,kw OR (Cardiotonic):ti,ab,kw | 1561 |
| 29 | #25 OR #26 OR #27 OR #28 | 2118 |
| 30 | #5 OR #11 OR #12 OR #13 OR #14 OR #15 OR #20 OR #24 OR #29 | 14349 |
| 31 | #1 AND #2 AND #30 | 74 |
| **ClinicalTrials.gov** | | |
| 1 | (ST Elevation Myocardial Infarction OR STEMI OR ST Segment Elevation Myocardial Infarction OR ST Elevated Myocardial Infarction) AND (Shock, Cardiogenic OR Cardiogenic Shock) AND (intra aortic balloon pumping OR intra aortic balloon pumping OR intraaortic balloon pumping OR balloon pumping, intraaortic OR pumping, intraaortic balloon OR pumping, intra aortic balloon OR balloon pumping, intra aortic OR pumping, intra aortic balloon OR extracorporeal membrane oxygenation OR extracorporeal membrane oxygenations OR membrane oxygenation, extracorporeal OR ecmo extracorporeal membrane oxygenation OR extracorporeal life support OR extracorporeal life supports OR life support, extracorporeal OR ecmo treatment OR ecmo treatments OR treatment, ecmo OR ecls treatment OR ecls treatments OR treatment, ecls OR oxygenation, extracorporeal membrane OR venoarterial ecmo OR ecmo, venoarterial OR venoarterial ecmos OR venoarterial extracorporeal membrane oxygenation OR venovenous ecmo OR ecmo, venovenous OR venovenous ecmos OR venovenous extracorporeal membrane oxygenation OR Impella OR Tandem Heart OR Protek Duo OR Centrimag OR vasoconstrictor agents OR agents, vasoconstrictor OR vasopressor agent OR agent, vasopressor OR vasoactive agonists OR agonists, vasoactive OR vasopressor agents OR agents, vasopressor OR vasoconstrictors OR vasoconstrictor drugs OR drugs, vasoconstrictor OR vasoconstrictor agent OR agent, vasoconstrictor OR vasoactive agonist OR agonist, vasoactive OR vasoconstrictor OR vasoconstrictor drug OR drug, vasoconstrictor OR vasodilator agents OR agents, vasodilator OR vasodilator drugs OR drugs, vasodilator OR vasodilators OR vasorelaxants OR vasodilator OR vasodilator agent OR agent, vasodilator OR vasodilator drug OR drug, vasodilator OR vasorelaxant OR vasoactive antagonists OR antagonists, vasoactive OR cardiotonic agents OR cardiotonic agent OR agent, cardiotonic OR myocardial stimulants OR cardiac stimulants OR cardioprotective agents OR cardiotonic drugs OR cardiotonics OR inotropic agents, positive cardiac OR cardiac stimulant OR stimulant, cardiac OR cardiotonic drug OR drug, cardiotonic OR cardiotonic OR cardioprotective agent OR agent, cardioprotective OR myocardial stimulant OR stimulant, myocardial) | 24 |
| **ICTRP** | | |
| 1 | (ST Elevation Myocardial Infarction OR STEMI OR ST Segment Elevation Myocardial Infarction OR ST Elevated Myocardial Infarction) AND (Shock, Cardiogenic OR Cardiogenic Shock) AND (intra aortic balloon pumping OR intra aortic balloon pumping OR intraaortic balloon pumping OR balloon pumping, intraaortic OR pumping, intraaortic balloon OR pumping, intra aortic balloon OR balloon pumping, intra aortic OR pumping, intra aortic balloon OR extracorporeal membrane oxygenation OR extracorporeal membrane oxygenations OR membrane oxygenation, extracorporeal OR ecmo extracorporeal membrane oxygenation OR extracorporeal life support OR extracorporeal life supports OR life support, extracorporeal OR ecmo treatment OR ecmo treatments OR treatment, ecmo OR ecls treatment OR ecls treatments OR treatment, ecls OR oxygenation, extracorporeal membrane OR venoarterial ecmo OR ecmo, venoarterial OR venoarterial ecmos OR venoarterial extracorporeal membrane oxygenation OR venovenous ecmo OR ecmo, venovenous OR venovenous ecmos OR venovenous extracorporeal membrane oxygenation OR Impella OR Tandem Heart OR Protek Duo OR Centrimag OR vasoconstrictor agents OR agents, vasoconstrictor OR vasopressor agent OR agent, vasopressor OR vasoactive agonists OR agonists, vasoactive OR vasopressor agents OR agents, vasopressor OR vasoconstrictors OR vasoconstrictor drugs OR drugs, vasoconstrictor OR vasoconstrictor agent OR agent, vasoconstrictor OR vasoactive agonist OR agonist, vasoactive OR vasoconstrictor OR vasoconstrictor drug OR drug, vasoconstrictor OR vasodilator agents OR agents, vasodilator OR vasodilator drugs OR drugs, vasodilator OR vasodilators OR vasorelaxants OR vasodilator OR vasodilator agent OR agent, vasodilator OR vasodilator drug OR drug, vasodilator OR vasorelaxant OR vasoactive antagonists OR antagonists, vasoactive OR cardiotonic agents OR cardiotonic agent OR agent, cardiotonic OR myocardial stimulants OR cardiac stimulants OR cardioprotective agents OR cardiotonic drugs OR cardiotonics OR inotropic agents, positive cardiac OR cardiac stimulant OR stimulant, cardiac OR cardiotonic drug OR drug, cardiotonic OR cardiotonic OR cardioprotective agent OR agent, cardioprotective OR myocardial stimulant OR stimulant, myocardial) | 6 |

# Supplemental Table S2 Baseline Characteristics of Studies Incorporated in the Meta-Analysis

| **Study ID**  **(first author)** | **Sample**  **size** | **Study**  **design** | **Treat**  **ment1** | **Treat**  **ment2** | **Mortality** | **Other**  **outcomes** | **Mean**  **age** | **Male**  **sex** | **Location** | |
| --- | --- | --- | --- | --- | --- | --- | --- | --- | --- | --- |
| Møller, J. E. | 355 | RCT | Impella | Control | 30 days | A B | 68 | 79.2% | Denmark, Germany, and the United Kingdom | |
| Diaz-Arocutipa, Carlos* | 87480 | CS | NA | NA | in-hospital |  | 67 | 66.6% | the United States  (multicenter) | |
| A. Somaschini | 168 | CS | IABP | Control | 30 days |  | NS | 69.6% | Italy (single center) | |
| Pahuja, M.* | 167813 | CS | NA | NA | in-hospital |  | 67 | 63.6% | the United States  (multicenter) | |
| Lan, W. R. | 79 | CS | IABP | Control | in-hospital |  | 76 | 74.7% | China (single center) | |
| Petrovic, Milovan | 470** | CS | IABP | Control | in-hospital |  | 69 | 58.3% | Serbia (single center) | |
| Boudiche, S. | 122 | CS | IABP | Control | 30 days |  | 65 | 75.4% | Tunis (single center) | |
| R. de la Espriella-Juan | 73 | CS | IABP | Control | 30 days | A B | 70 | 57.5% | Spain (single center) | |
| M. B. Iqbal | 278 | CS | IABP | Control | 30 days | C | NS | 72.3% | Canada (multicenter) | |
| J. K. Jensen | 286 | CS | IABP | Control | 30 days | C | 71 | 70.3% | Denmark  (single center) | |
| Pathak, R. | 37868 | CS | IABP | Control | in-hospital |  | NS | NS | the United States  (multicenter) | |
| Dziewierz, Artur | 51 | CS | IABP | Control | 30 days | A C | 68 | 64.7% | Finland, Germany, Italy, Poland, Slovenia, Spain, Sweden | |
| K. D. Sjauw | 292 | CS | IABP | Control | 30 days | A B | 64 | 67.7% | the Netherlands  (single center) | |
| Gu, J. | 91 | CS | IABP | Control | 30 days | A | 69 | 63.7% | China (single center) | |
| Briasoulis, A.* | 42892 | CS | NA | NA | in-hospital | B D E | 67 | 65.9% | the United States  (multicenter) | |
| Singh, Sanjeet Singh Avtaar | 23 | CS | ECMO | Control | 30 days |  | 50 | 65.2% | the United Kingdom  (single center) | |
| Thakkar, Samarthkumar | 14690 | CS | IABP | Impella | in-hospital | A B D E | 65 | 72.9% | the United States  (multicenter) | |
| Ouweneel, Dagmar M. | 48 | RCT | IABP | Impella | 30 days | A B | 59 | 79.2% | the Netherlands  (multicenter) | |
| Sheu, Jiunn-Jye | 71 | CS | IABP | IABP+  ECMO | 30 days |  | 66 | NS | China (single center) | |
| Pan, Y. | 48 | CS | rhBNP+IABP | IABP | in-hospital |  | 65 | 72.9% | China (single center) | |
| Yahagi, Kazuyuki | 1861 | CS | Impella | ECMO+Impella | in-hospital |  | NS | NS | Japan (multicenter) | |
| Yang, Hongbo | 5176 | CS | IABP | ECMO | in-hospital |  | 62 | 78.7% | China (multicenter) | |
| A = Bleeding; B = Stroke; C = 1-year mortality; D = Acute kidney injury (AKI); E = AKI requiring hemodialysis; CS = cohort study; NA = not applicable; NS = not specified; RCT = randomized controlled trial.  *Studies assess multiple treatments. **This sample size is inflated due to the employment of standardized rate adjustments. | | | | | | | | | |  |

# Supplemental Table S3 Demographic Profiles of the Meta-Analyzed Study Populations

| **Study ID**  **(first author)** | **Treat**  **ment1** | **Treat**  **ment2** | **Smoking** | **Hyper**  **tension** | **Diabetes** | **Hyper/dys**  **lipidemia** | **Mechanical**  **ventilation** | **Anterior**  **Myocardial**  **infarction** | **Ejection**  **fraction (EF) mean** |
| --- | --- | --- | --- | --- | --- | --- | --- | --- | --- |
|  |  |  |  |  |  |  |  |  |  |
| Møller, J. E. | Impella | Control | NS | 49.7/53.4 | 18.4/26.7 | NS | 74.3/65.9 | 70.4/73.3 | (Median)  25/25 |
| Diaz-Arocutipa, Carlos* | NA | NA | NS | 69.1 | 35.8 | 53.0 | 48.7 | NS | NS |
| A. Somaschini | IABP | Control | NS | NS | 26.2/13.1 | NS | NS | 66.7/54.8 | NS |
| Pahuja, M.* | NA | NA | NS | 52.0 | 29.8 | 39.3 | NS | NS | NS |
| Lan, W. R. | IABP | Control | 40.0/38.0 | 70.0/65.5 | 40.0/44.8 | 18.0/17.2 | NS | NS | 46.7/53.9 |
| Petrovic, Milovan | IABP | Control | 31.9 | 58.3 | 27.2 | 18.7 | 100.0 | 54.0 | 36.0 |
| Boudiche, S. | IABP | Control | 70.5 | 41.0 | 49.2 | 18.0 | 10.7 | 58.2 | 31.5 |
| R. de la Espriella-Juan | IABP | Control | 47.7/48.3 | 56.8/79.3 | 34.1/51.7 | 45.5/44.8 | 53.4 | 70.5/58.6 | 31/37 |
| M. B. Iqbal | IABP | Control | 23.0/25.6 | 49.6/45.7 | 27.9/25.2 | 31.0/31.3 | NS | NS | (EF<30%)  27.4/27.8 |
| J. K. Jensen | IABP | Control | 39.6/54.4 | 29.9/32.1 | 11.3/13.5 | 32.2/21.6 | NS | 69.1/52.4 | NS |
| Pathak, R. | IABP | Control | NS | NS | NS | NS | NS | NS | NS |
| Dziewierz, Artur | IABP | Control | 26.7/23.8 | NS | 26.7/4.8 | NS | NS | NS | NS |
| K. D. Sjauw | IABP | Control | 36.2/52.7 | 34.7/31.2 | 18.1/17.2 | 21.1/34.4 | NS | NS | NS |
| Gu, J. | IABP | Control | 55.8/33.3 | 69.8/66.7 | 39.5/35.4 | 32.6/37.5 | 60.5/56.3 | NS | NS |
| Briasoulis, A.* | NA | NA | NS | 71.1 | 36.6 | NS | NS | NS | NS |
| Singh, Sanjeet Singh Avtaar | ECMO | Control | 34.8 | 13.0 | 4.3 | NS | NS | NS | 35.6 |
| Thakkar, Samarthkumar | IABP | Impella | 23.9/24.9 | 30.1/30.6 | 12.1/12.9 | 47.2/48.2 | NS | NS | NS |
| Ouweneel, Dagmar M. | IABP | Impella | 31.6/61.1 | 28.6/20.0 | 13.0/9.1 | 23.8/20.0 | 100/100 | NS | 49/46 |
| Sheu, Jiunn-Jye | IABP | IABP+  ECMO | NS | NS | NS | NS | 76.0/84.8 | 44.0/80.4 | NS |
| Pan, Y. | rhBNP+IABP | IABP | 56.0/52.2 | 48.0/65.2 | 36.0/30.4 | NS | 48.0/52.2 | 1.0 | 38.6/39.6 |
| Yahagi, Kazuyuki | Impella | ECMO+  Impella | NS | NS | NS | NS | NS | NS | NS |
| Yang, Hongbo | IABP | ECMO | 37.7/38.8 | 46.7/46.2 | 23.1/22.1 | 20.9/20.5 | NS | NS | NS |
| All values are presented as ‘treatment1/treatment2’ or as totals with percentiles, with EF reported as the mean instead (or as otherwise specified).  ECMO = extracorporeal membrane oxygenation; IABP = intra-aortic balloon pump; NA = not applicable; NS = not specified; rhBNP = recombinant human B-type natriuretic peptide.  *Studies assess multiple treatments. | | | | | | | | | |

# Supplemental Table S4 Angiographic Profiles of the Meta-Analyzed Study Populations

| **Study ID**  **(first author)** | **Treat**  **ment1** | **Treat**  **ment2** | **Infarct related artery** | | | | **Post-procedural TIMI Flow <3** | **Number of diseased vessels** | | |
| --- | --- | --- | --- | --- | --- | --- | --- | --- | --- | --- |
|  |  |  | **LMCA** | **LAD** | **LCX** | **RCA** |  | **1** | **2** | **3 or more** |
| Møller, J. E. | Impella | Control | NS | NS | NS | NS | NS | 29.1/26.7^ | 39.1/36.4 | 31.3/36.9 |
| Diaz-Arocutipa, Carlos* | NA | NA | NS | NS | NS | NS | NS | NS | NS | NS |
| A. Somaschini | IABP | Control | 7.1/2.4 | NS | NS | NS | 23.8/20.2 | NS | NS | 38.1/31.0 |
| Pahuja, M.* | NA | NA | NS | NS | NS | NS | NS | NS | NS | NS |
| Lan, W. R. | IABP | Control | NS | NS | NS | NS | NS | 28.0/20.7 | 26.0/31.0 | 46.0/48.3 |
| Petrovic, Milovan | IABP | Control | NS | NS | NS | NS | 23.0 | 27.7 | NS | NS |
| Boudiche, S. | IABP | Control | 0.8 | 57.4 | 12.3 | 32.0 | 25.4 | 35.2 | 31.1 | 33.6 |
| R. de la Espriella-Juan | IABP | Control | 27.3/3.4 | 47.7/55.2 | 13.6/31.0 | 11.4/10.3 | 18.2/17.2 | 38.6/51.7 | 34.1/24.1 | 27.3/24.1 |
| M. B. Iqbal | IABP | Control | 10.8/10.1 | 82.0/79.1 | 62.6/59.0 | 70.5/70.5 | nearly 0? | 25.2/24.5 | NS | NS |
| J. K. Jensen | IABP | Control | 31.9/17.0 | 42.0/39.3 | 7.2/12.1 | 18.8/31.3 | 22.5/28.4 | 40.8/34.0 | NS | NS |
| Pathak, R. | IABP | Control | NS | NS | NS | NS | NS | NS | NS | NS |
| Dziewierz, Artur | IABP | Control | NS | 66.7/38.1 | NS | NS | 20.0/23.8 | 33.3/23.8 | NS | NS |
| K. D. Sjauw | IABP | Control | NS | 59.8/28.0 | NS | NS | 32.2/25.0 | 37.2/59.8 | NS | NS |
| Gu, J. | IABP | Control | 2.3/4.2 | 65.1/56.3 | 14.0/14.6 | 20.9/25.0 | NS | 25.6/25.0 | 23.3/29.2 | 51.2/45.8 |
| Briasoulis, A.* | NA | NA | NS | NS | NS | NS | NS | NS | NS | NS |
| Singh, Sanjeet Singh Avtaar | ECMO | Control | 8.7 | 39.1 | 4.3 | 26.1 | NS | 78.3 | NS | NS |
| Thakkar, Samarthkumar | IABP | Impella | NS | NS | NS | NS | NS | NS | NS | NS |
| Ouweneel, Dagmar M. | IABP | Impella | 8.3/4.2 | 62.5/66.7 | 12.5/25.0 | 16.7/4.2 | (TIMI 0 or 1) 0/4.2 | 12.5/37.5 | NS | NS |
| Sheu, Jiunn-Jye | IABP | IABP+  ECMO | 12.0/28.3 | 80.0/87.0 | 56.0/60.9 | 84.0/65.2 | 44.0/19.6 | 24.0/17.4 | NS | NS |
| Pan, Y. | rhBNP+IABP | IABP | 16.0/17.4 | 84.0/78.3 | 0/4.3 | NS | 0/0 | NS | NS | NS |
| Yahagi, Kazuyuki | Impella | ECMO+Impella | NS | NS | NS | NS | NS | NS | NS | NS |
| Yang, Hongbo | IABP | ECMO | 9.1/7.4 | 62.7/61.7 | 24.1/23.5 | 55.3/56.7 | NS | NS | NS | NS |
| All values are presented as ‘treatment1/treatment2’ or as totals with percentiles. ECMO = extracorporeal membrane oxygenation; IABP = intra-aortic balloon pump; LAD = left anterior descending; LCX = left circumflex; LMCA = left main coronary artery; NA = not applicable; NS = not specified; RCA = right coronary artery; rhBNP = recombinant human B-type natriuretic peptide; TIMI = thrombolysis in myocardial infarction.  *Studies assess multiple treatments. ^ No diseased vessels were observed on coronary angiography (CAG) in one case. ? Values may be erroneously characterized as ‘nearly 100’ in the original article. | | | | | | | | | | |

# Supplemental Table S5 Rate Standardization in Study Petrovic, Milovan 2019

| **Crude** | **IABP** | | | **No IABP** | | | |
| --- | --- | --- | --- | --- | --- | --- | --- |
|  | **Dead** | **Total** | **Rate** | **Dead** | **Total** | | **Rate** |
| Single vessel | 13 | 21 | 61.9% | 24 | 44 | | 54.55% |
| MVD with CR | 5 | 14 | 35.71% | 7 | 14 | | 50% |
| MVD with culprit revascularization | 38 | 49 | 77.55% | 60 | 93 | | 64.52% |
|  | | | | | | | |
| **Standardized** | **IABP** | | **No IABP** | | | **Std. total** | |
|  | **Dead** | **Rate** | **Dead** | **Rate** | |  |  |
| Single vessel | 40 | 61.90% | 35 | 54.55% | | 65 | |
| MVD with CR | 10 | 35.71% | 14 | 50% | | 28 | |
| MVD with culprit revascularization | 110 | 77.55% | 92 | 64.52% | | 142 | |
| **Sum** | **160** |  | **141** |  | | **235** | |
| CR = complete revascularization; IABP = intra-aortic balloon pump; MVD = multivessel disease; Std. total = total number of patients after rate standardization.  In this study, the IABP group contained a larger proportion of patients with multivessel disease than the no-IABP group (75.0% vs. 70.9%). Without adjustment, this imbalance would bias the comparison: the higher overall mortality in the IABP group could simply reflect its greater burden of multivessel disease rather than a true difference attributable to IABP use. Such a comparison would clearly be unreasonable.  Rate standardization was performed to account for differences in the distribution of coronary disease complexity between groups. The standard population (Std. total) for each coronary disease category (single-vessel disease, MVD with CR, MVD with culprit revascularization) was defined as the total number of patients pooled across IABP and no-IABP groups within that category. For each group, the crude mortality rate within a given category was multiplied by the corresponding standard population to obtain the standardized number of deaths. This procedure was repeated for all three coronary disease categories. The standardized numbers of deaths and total patients were then summed across categories to yield overall standardized totals of 235 patients per group, with 160 deaths in the IABP group and 141 deaths in the no-IABP group. | | | | | | | |

# Supplemental Table S6 GRADE Evidence Assessment for Pooled Analyses

| **Outcome** | **Treat**  **ment1** | **Treat**  **ment2** | **Direct Evidence** | | **Indirect Evidence** | | **Synthesized Evidence** | | **p value** |
| --- | --- | --- | --- | --- | --- | --- | --- | --- | --- |
|  |  |  | **OR (95% CrI/CI)** | **Quality** | **OR (95% CrI)** | **Quality*** | **OR (95% CrI)** | **Quality**** |  |
| **Network meta-analysis** | | | | | | | | | |
| Short-term mortality | ECMO | Control | 1.4 (0.52, 3.9) | Very low^a,c^ | 1.4 (0.42, 4.4) | Very low | 1.32 (0.62, 2.81) | Very low | 0.970 |
|  | IABP | Control | 1.1 (0.67, 1.7) | Very low^a^ | 0.58 (0.12, 3.2) | Very low | 1.04 (0.67, 1.64) | Very low | 0.478 |
|  | Impella | Control | 1.5 (0.46, 4.7) | Very low^a,d^ | 1.3 (0.34, 5.1) | Very low | 1.55 (0.67, 3.46) | Very low | 0.910 |
|  | IABP | ECMO | 0.75 (0.33, 1.7) | Very low^a^ | 0.53 (0.013, 11.) | Very low | 0.79 (0.39, 1.61) | Very low | 0.837 |
|  | Impella | ECMO | 3.6 (0.84, 15.) | Moderate^b^ | 0.57 (0.17, 1.8) | Very low | 1.17 (0.43, 3.08) | Moderate | 0.052 |
|  | Impella | IABP | 1.4 (0.57, 3.5) | Very low^a,c,d^ | 0.75 (0.15, 3.7) | Very low | 1.48 (0.66, 3.22) | Very low | 0.464 |
| Bleeding | IABP | Control | 1.8 (0.67, 6.) | Low | 1.0 (0.093, 6.3) | Very low | 1.49 (0.72, 3.66) | Low | 0.501 |
|  | Impella | Control | 2.1 (0.39, 11.) | Moderate^b^ | 3.6 (0.95, 28.) | Low^b^ | 2.55 (1.18, 8.51) | Moderate | 0.509 |
|  | Impella | IABP | 1.9 (0.73, 9.5) | Low^d^ | 1.2 (0.14, 7.8) | Very low | 1.68 (0.83, 4.68) | Low | 0.506 |
| Stroke | IABP | Control | 1.0 (0.32, 3.3) | Low | 1.2 (0.13, 13.) | Very low | 1.16 (0.38, 3.59) | Low | 0.881 |
|  | Impella | Control | 3.8 (0.91, 10.) | Moderate^b,d^ | 1.8 (0.15, 25.) | Very low | 2.78 (0.85, 8.1) | Moderate | 0.600 |
|  | Impella | IABP | 2.5 (0.69, 7.9) | Low^a,b,c,d^ | 1.6 (0.060, 38.) | Very low | 2.4 (0.82, 6.25) | Low | 0.797 |
| **Traditional meta-analysis** | | | | | | | | | |
| Short-term mortality | ECMO | Control | 2.42 (2.25, 2.61) | Moderate^b,c^ | -- | -- | -- | -- | -- |
|  | IABP | Control | 1.03 (0.73, 1.45) | Very low^a^ | -- | -- | -- | -- | -- |
|  | Impella | Control | 1.47 (0.41, 5.22) | Very low^a,d^ | -- | -- | -- | -- | -- |
|  | IABP | ECMO | 0.75 (0.28, 2.03) | Very low^a^ | -- | -- | -- | -- | -- |
|  | Impella | IABP | 1.51 (1.10, 2.06) | Low^c,d^ | -- | -- | -- | -- | -- |
| Bleeding | IABP | Control | 1.56 (0.91, 2.68) | Low | -- | -- | -- | -- | -- |
|  | Impella | IABP | 2.21 (0.78, 6.31) | Moderate^b^ | -- | -- | -- | -- | -- |
| Stroke | IABP | Control | 1.00 (0.84, 1.18) | Low | -- | -- | -- | -- | -- |
|  | Impella | Control | 3.44 (1.56, 7.56) | Moderate^b^ | -- | -- | -- | -- | -- |
|  | Impella | IABP | 1.62 (1.38, 1.91) | Low^c^ | -- | -- | -- | -- | -- |
| 1-year mortality | IABP | Control | 1.16 (0.64, 2.10) | Low^c^ | -- | -- | -- | -- | -- |
| AKI | Impella | IABP | 1.55 (1.10, 2.18) | Very low^a^ | -- | -- | -- | -- | -- |
| AKI requiring hemodialysis | Impella | IABP | 1.67 (1.50, 1.87) | Low | -- | -- | -- | -- | -- |
| AKI = acute kidney injury; CI = confidence interval; CrI = credible interval; ECMO = extracorporeal membrane oxygenation; IABP = intra-aortic balloon pump; OR = odds ratio a^-^. Serious inconsistency: notable heterogeneity was observed among studies. b^+^. Strong association observed. c. One study was excluded following influential analysis detecting heterogeneity in traditional meta-analysis. d. This comparison encompassed evidence from a randomized controlled trial. + This will upgrade the quality of evidence. - This will downgrade the quality of evidence. * The quality of indirect evidence is limited due to its non-primary provenance. ** Synthesis evidence quality is determined by the higher evidentiary tier when direct and indirect ones diverge. | | | | | | | | | |

# Supplemental Table S7 League Table of Network Meta-analysis

| Control | 1.32 (0.62, 2.81) | **6.06 (1.03, 35.28)** | 1.04 (0.67, 1.64) | 0.25 (0.04, 1.68) | 1.55 (0.67, 3.46) | 0.61 (0.08, 4.64) |
| --- | --- | --- | --- | --- | --- | --- |
| 0.76 (0.36, 1.61) | ECMO | 4.61 (0.72, 28.69) | 0.79 (0.39, 1.61) | 0.19 (0.02, 1.39) | 1.17 (0.43, 3.08) | 0.47 (0.06, 3.82) |
| **0.17 (0.03, 0.97)** | 0.22 (0.03, 1.38) | ECMO + Impella | **0.17 (0.03, 1)** | **0.04 (0, 0.53)** | 0.25 (0.05, 1.22) | 0.1 (0.01, 1.44) |
| 0.96 (0.61, 1.49) | 1.26 (0.62, 2.57) | **5.81 (1, 33.53)** | IABP | 0.24 (0.04, 1.51) | 1.48 (0.66, 3.22) | 0.59 (0.08, 4.22) |
| 4.01 (0.6, 28.27) | 5.29 (0.72, 40.94) | **24.36 (1.88, 318.56)** | 4.2 (0.66, 28.36) | IABP + ECMO | 6.21 (0.83, 48.23) | 2.48 (0.16, 37.86) |
| 0.65 (0.29, 1.48) | 0.85 (0.32, 2.31) | 3.92 (0.82, 18.81) | 0.67 (0.31, 1.5) | 0.16 (0.02, 1.21) | Impella | 0.4 (0.05, 3.37) |
| 1.63 (0.22, 12.41) | 2.14 (0.26, 17.86) | 9.93 (0.69, 139.47) | 1.69 (0.24, 12.45) | 0.4 (0.03, 6.23) | 2.52 (0.3, 20.91) | rhBNP + IABP |
|  |  |  |  |  |  |  |
| Control | 1.49 (0.72, 3.66) | **2.55 (1.18, 8.51)** |  | Control | 1.16 (0.38, 3.59) | 2.78 (0.85, 8.1) |
| 0.67 (0.27, 1.39) | IABP | 1.68 (0.83, 4.68) |  | 0.86 (0.28, 2.64) | IABP | 2.4 (0.82, 6.25) |
| **0.39 (0.12, 0.84)** | 0.6 (0.21, 1.2) | Impella |  | 0.36 (0.12, 1.17) | 0.42 (0.16, 1.23) | Impella |
| Odds ratios (ORs) with 95% credible intervals are calculated. Values > 1.00 indicate higher odds of the outcome in the column group compared to the row group (column vs. row). | | | | | | |
|  | | | | | | |

# Supplemental Table S8 A Brief Overview Comparing MCS

| **Device** | **CO Increase** | **LV Unloading** | **Key Advantages** | **Key Disadvantages** |
| --- | --- | --- | --- | --- |
| **Impella (2.5/CP**  **/5.0/5.5)** | +1.5-5.5 L/min | Direct (active LV drainage) | Boosting CO and greatly unloads LV | Pricey, requires adequate heart function, bleeding, hemolysis and renal injury risks |
| **IABP** | +0.5-1.0 L/min | Indirect (reduce LV afterload) | Mildly elevates CO, easy to implant, low cost | Minimal CO support, no direct LV unloading |
| **ECMO** | +4-7 L/min | None (increase LV afterload) | Full cardiopulmonary support | Requires LV venting such as IABP/Impella |
| **ECPELLA (ECMO + Impella)** | Combined  effect | Combined effect | Maximizes CO + LV unloading (particularly effective in severe cardiogenic shock) | Complex setup, exorbitant price, elevated risks of dual-device complications vs. IABP |
| **ECMO + IABP** | Combined  effect | Combined effect | Substantially augments CO, lower risks of complications vs. Impella | Insufficient for severe shock, dual-device complications |
| CO = cardiac output; ECMO = extracorporeal membrane oxygenation; IABP = intra-aortic balloon pump; LV = left ventricle. | | | | |

# Database-by-year Overlap Matrix

| **Database** | **Recruitment periods** | **Study** |
| --- | --- | --- |
| National Inpatient Sample (NIS) database of the US | 2004-2011 | Pathak, R. n=37868 IABP, control |
| National Inpatient Sample (NIS) database of the US | 2005-2014 | Pahuja, M. n=167813 IABP, ECMO, control |
| National Inpatient Sample (NIS) database | October 2015-December 2018 | Thakkar, Samarthkumar n=14690 IABP, Impella |
| National Inpatient Sample (NIS) database of the US | 2016–2019 | Diaz-Arocutipa, Carlos n=87480 IABP, ECMO, control |
| National Readmission Database (NRD) of the US | 2016-2019 | Briasoulis, A. n=42892 IABP, ECMO, Impella, control |
| Chang Gung Memorial Hospital-Kaohsiung Medical Center | May 1993-July 2002 | Sheu, Jiunn-Jye n=71 |
| Academic Medical Center-University of Amsterdam | January 1997-March 2005 | K. D. Sjauw n=292 |
| Odense University Hospital | January 1, 2002-December 31, 2010 | J. K. Jensen n=286 |
| Min Hang Hospital | January 2003-December 2008 | Gu, J. n=91 |
| Policlinico San Matteo | 1 January 2005-30 June 2018 | A. Somaschini n=168 |
| The EUROTRANSFER Registry | November 2005-January 2007 | Dziewierz, Artur n=51 |
| Institute of Cardiovascular Diseases of Vojvodina | August 2007-October 2016 | Petrovic, Milovan n=470 |
| British Columbia Cardiac Registry | 2008–2013 | M. B. Iqbal n=278 |
| Consorcio Hospital General Universitario de Valencia | January 2009-August 2015 | R. de la Espriella-Juan n=73 |
| Golden Jubilee National Hospital | January 2009-August 2017 | Singh, Sanjeet Singh Avtaar n=23 |
| Rabta Hospital | January 2009-August 2018 | Boudiche, S. n=122 |
| Shanghai 6th People’s Hospital | March 2009-March 2013 | Pan, Y. n=48 |
| MacKay Memorial Hospital | January 2012-December 2015 | Lan, W. R. n=79 |
| Chinese Cardiovascular Association database | January 1, 2019-December 31, 2021 | Yang, Hongbo n=5176 |
| The J-PVAD registry | February 2020-December 2021 | Yahagi, Kazuyuki n=1861 |
| Randomized controlled trials preclude overlap by definition. | | |

# Supplemental Figure S1 Primary Outcome Comparative Evidence Network

The network diagram utilizes proportional visualization to represent research evidence: nodal diameters scale with patient cohort sizes across studies for each intervention, while edge thickness indicates the quantity of head-to-head comparative trials between treatment pairs. ECMO = extracorporeal membrane oxygenation; IABP = intra-aortic balloon pump; rhBNP = recombinant human B-type natriuretic peptide.

# Supplemental Figure S2 Secondary Outcomes Comparative Evidence Network

# Newcastle - Ottawa Quality Assessment Scale for Cohort Studies

Note: A study can be awarded a maximum of one star for each numbered item within the Selection and Outcome categories. A maximum of two stars can be given for Comparability

**Study: Singh, Sanjeet Singh Avtaar et al. 2019**

| Selection | 1) Representativeness of the exposed cohort | 0 |
| --- | --- | --- |
|  | 2) Selection of the non-exposed cohort | 1 |
|  | 3) Ascertainment of exposure | 1 |
|  | 4) Demonstration that the outcome of interest was not present at the start of the study | 1 |
| Comparability | 1) Comparability of cohorts on the basis of the design or analysis | 1 |
| Outcome | 1) Assessment of outcome | 1 |
|  | 2) Was follow-up long enough for outcomes to occur | 1 |
|  | 3) Adequacy of follow-up of cohorts | 1 |
| Total Score | 3/4+1/2+3/3 | 7/9 |

**Study: Pahuja, M. et al. 2021**

| Selection | 1) Representativeness of the exposed cohort | 1 |
| --- | --- | --- |
|  | 2) Selection of the non-exposed cohort | 1 |
|  | 3) Ascertainment of exposure | 1 |
|  | 4) Demonstration that the outcome of interest was not present at the start of the study | 1 |
| Comparability | 1) Comparability of cohorts on the basis of the design or analysis | 1 |
| Outcome | 1) Assessment of outcome | 1 |
|  | 2) Was follow-up long enough for outcomes to occur | 1 |
|  | 3) Adequacy of follow-up of cohorts | 1 |
| Total Score | 4/4+1/2+3/3 | 8/9 |

**Study: Diaz-Arocutipa, Carlos et al. 2025**

| Selection | 1) Representativeness of the exposed cohort | 1 |
| --- | --- | --- |
|  | 2) Selection of the non-exposed cohort | 1 |
|  | 3) Ascertainment of exposure | 1 |
|  | 4) Demonstration that the outcome of interest was not present at the start of the study | 1 |
| Comparability | 1) Comparability of cohorts on the basis of the design or analysis | 1 |
| Outcome | 1) Assessment of outcome | 1 |
|  | 2) Was follow-up long enough for outcomes to occur | 1 |
|  | 3) Adequacy of follow-up of cohorts | 1 |
| Total Score | 4/4+1/2+3/3 | 8/9 |

**Study: Briasoulis, A. et al. 2023**

| Selection | 1) Representativeness of the exposed cohort | 1 |
| --- | --- | --- |
|  | 2) Selection of the non-exposed cohort | 1 |
|  | 3) Ascertainment of exposure | 1 |
|  | 4) Demonstration that the outcome of interest was not present at the start of the study | 1 |
| Comparability | 1) Comparability of cohorts on the basis of the design or analysis | 0 |
| Outcome | 1) Assessment of outcome | 1 |
|  | 2) Was follow-up long enough for outcomes to occur | 1 |
|  | 3) Adequacy of follow-up of cohorts | 1 |
| Total Score | 4/4+0/2+3/3 | 7/9 |

**Study: Gu, J. et al.	2010**

| Selection | 1) Representativeness of the exposed cohort | 0 |
| --- | --- | --- |
|  | 2) Selection of the non-exposed cohort | 1 |
|  | 3) Ascertainment of exposure | 1 |
|  | 4) Demonstration that the outcome of interest was not present at the start of the study | 1 |
| Comparability | 1) Comparability of cohorts on the basis of the design or analysis | 2 |
| Outcome | 1) Assessment of outcome | 1 |
|  | 2) Was follow-up long enough for outcomes to occur | 1 |
|  | 3) Adequacy of follow-up of cohorts | 1 |
| Total Score | 3/4+2/2+3/3 | 8/9 |

**Study: Dziewierz, Artur et al. 2014**

| Selection | 1) Representativeness of the exposed cohort | 1 |
| --- | --- | --- |
|  | 2) Selection of the non-exposed cohort | 1 |
|  | 3) Ascertainment of exposure | 1 |
|  | 4) Demonstration that the outcome of interest was not present at the start of the study | 1 |
| Comparability | 1) Comparability of cohorts on the basis of the design or analysis | 1 |
| Outcome | 1) Assessment of outcome | 1 |
|  | 2) Was follow-up long enough for outcomes to occur | 1 |
|  | 3) Adequacy of follow-up of cohorts | 1 |
| Total Score | 4/4+1/2+3/3 | 8/9 |

**Study: Pathak, R. et al. 2014**

| Selection | 1) Representativeness of the exposed cohort | 1 |
| --- | --- | --- |
|  | 2) Selection of the non-exposed cohort | 1 |
|  | 3) Ascertainment of exposure | 1 |
|  | 4) Demonstration that the outcome of interest was not present at the start of the study | 1 |
| Comparability | 1) Comparability of cohorts on the basis of the design or analysis | 1 |
| Outcome | 1) Assessment of outcome | 1 |
|  | 2) Was follow-up long enough for outcomes to occur | 1 |
|  | 3) Adequacy of follow-up of cohorts | 1 |
| Total Score | 4/4+1/2+3/3 | 8/9 |

**Study: Lan, W. R. et al. 2021**

| Selection | 1) Representativeness of the exposed cohort | 0 |
| --- | --- | --- |
|  | 2) Selection of the non-exposed cohort | 1 |
|  | 3) Ascertainment of exposure | 1 |
|  | 4) Demonstration that the outcome of interest was not present at the start of the study | 1 |
| Comparability | 1) Comparability of cohorts on the basis of the design or analysis | 1 |
| Outcome | 1) Assessment of outcome | 1 |
|  | 2) Was follow-up long enough for outcomes to occur | 1 |
|  | 3) Adequacy of follow-up of cohorts | 1 |
| Total Score | 3/4+1/2+3/3 | 7/9 |

**Study: Petrovic, Milovan et al. 2019**

| Selection | 1) Representativeness of the exposed cohort | 0 |
| --- | --- | --- |
|  | 2) Selection of the non-exposed cohort | 1 |
|  | 3) Ascertainment of exposure | 1 |
|  | 4) Demonstration that the outcome of interest was not present at the start of the study | 1 |
| Comparability | 1) Comparability of cohorts on the basis of the design or analysis | 1 |
| Outcome | 1) Assessment of outcome | 1 |
|  | 2) Was follow-up long enough for outcomes to occur | 1 |
|  | 3) Adequacy of follow-up of cohorts | 1 |
| Total Score | 3/4+1/2+3/3 | 7/9 |

**Study: Boudiche, S. et al. 2019**

| Selection | 1) Representativeness of the exposed cohort | 0 |
| --- | --- | --- |
|  | 2) Selection of the non-exposed cohort | 1 |
|  | 3) Ascertainment of exposure | 1 |
|  | 4) Demonstration that the outcome of interest was not present at the start of the study | 1 |
| Comparability | 1) Comparability of cohorts on the basis of the design or analysis | 1 |
| Outcome | 1) Assessment of outcome | 1 |
|  | 2) Was follow-up long enough for outcomes to occur | 1 |
|  | 3) Adequacy of follow-up of cohorts | 1 |
| Total Score | 3/4+1/2+3/3 | 7/9 |

**Study: R. de la Espriella-Juan et al. 2017**

| Selection | 1) Representativeness of the exposed cohort | 0 |
| --- | --- | --- |
|  | 2) Selection of the non-exposed cohort | 1 |
|  | 3) Ascertainment of exposure | 1 |
|  | 4) Demonstration that the outcome of interest was not present at the start of the study | 1 |
| Comparability | 1) Comparability of cohorts on the basis of the design or analysis | 2 |
| Outcome | 1) Assessment of outcome | 1 |
|  | 2) Was follow-up long enough for outcomes to occur | 1 |
|  | 3) Adequacy of follow-up of cohorts | 1 |
| Total Score | 3/4+2/2+3/3 | 8/9 |

**Study: K. D. Sjauw et al. 2012**

| Selection | 1) Representativeness of the exposed cohort | 0 |
| --- | --- | --- |
|  | 2) Selection of the non-exposed cohort | 1 |
|  | 3) Ascertainment of exposure | 1 |
|  | 4) Demonstration that the outcome of interest was not present at the start of the study | 1 |
| Comparability | 1) Comparability of cohorts on the basis of the design or analysis | 2 |
| Outcome | 1) Assessment of outcome | 1 |
|  | 2) Was follow-up long enough for outcomes to occur | 1 |
|  | 3) Adequacy of follow-up of cohorts | 1 |
| Total Score | 3/4+2/2+3/3 | 8/9 |

**Study: J. K. Jensen et al. 2015**

| Selection | 1) Representativeness of the exposed cohort | 0 |
| --- | --- | --- |
|  | 2) Selection of the non-exposed cohort | 1 |
|  | 3) Ascertainment of exposure | 1 |
|  | 4) Demonstration that the outcome of interest was not present at the start of the study | 1 |
| Comparability | 1) Comparability of cohorts on the basis of the design or analysis | 1 |
| Outcome | 1) Assessment of outcome | 1 |
|  | 2) Was follow-up long enough for outcomes to occur | 1 |
|  | 3) Adequacy of follow-up of cohorts | 1 |
| Total Score | 3/4+1/2+3/3 | 7/9 |

**Study: A. Somaschini et al. 2023**

| Selection | 1) Representativeness of the exposed cohort | 0 |
| --- | --- | --- |
|  | 2) Selection of the non-exposed cohort | 1 |
|  | 3) Ascertainment of exposure | 1 |
|  | 4) Demonstration that the outcome of interest was not present at the start of the study | 1 |
| Comparability | 1) Comparability of cohorts on the basis of the design or analysis | 2 |
| Outcome | 1) Assessment of outcome | 1 |
|  | 2) Was follow-up long enough for outcomes to occur | 1 |
|  | 3) Adequacy of follow-up of cohorts | 1 |
| Total Score | 3/4+2/2+3/3 | 8/9 |

**Study: M. B. Iqbal et al. 2016**

| Selection | 1) Representativeness of the exposed cohort | 1 |
| --- | --- | --- |
|  | 2) Selection of the non-exposed cohort | 1 |
|  | 3) Ascertainment of exposure | 1 |
|  | 4) Demonstration that the outcome of interest was not present at the start of the study | 1 |
| Comparability | 1) Comparability of cohorts on the basis of the design or analysis | 2 |
| Outcome | 1) Assessment of outcome | 1 |
|  | 2) Was follow-up long enough for outcomes to occur | 1 |
|  | 3) Adequacy of follow-up of cohorts | 1 |
| Total Score | 4/4+2/2+3/3 | 9/9 |

**Study: Yang, Hongbo et al. 2024**

| Selection | 1) Representativeness of the exposed cohort | 1 |
| --- | --- | --- |
|  | 2) Selection of the non-exposed cohort | 1 |
|  | 3) Ascertainment of exposure | 1 |
|  | 4) Demonstration that the outcome of interest was not present at the start of the study | 1 |
| Comparability | 1) Comparability of cohorts on the basis of the design or analysis | 2 |
| Outcome | 1) Assessment of outcome | 1 |
|  | 2) Was follow-up long enough for outcomes to occur | 1 |
|  | 3) Adequacy of follow-up of cohorts | 1 |
| Total Score | 4/4+2/2+3/3 | 9/9 |

**Study: Sheu, Jiunn-Jye et al. 2010**

| Selection | 1) Representativeness of the exposed cohort | 0 |
| --- | --- | --- |
|  | 2) Selection of the non-exposed cohort | 1 |
|  | 3) Ascertainment of exposure | 1 |
|  | 4) Demonstration that the outcome of interest was not present at the start of the study | 1 |
| Comparability | 1) Comparability of cohorts on the basis of the design or analysis | 1 |
| Outcome | 1) Assessment of outcome | 1 |
|  | 2) Was follow-up long enough for outcomes to occur | 1 |
|  | 3) Adequacy of follow-up of cohorts | 1 |
| Total Score | 3/4+1/2+3/3 | 7/9 |

**Study: Thakkar, Samarthkumar et al. 2021**

| Selection | 1) Representativeness of the exposed cohort | 1 |
| --- | --- | --- |
|  | 2) Selection of the non-exposed cohort | 1 |
|  | 3) Ascertainment of exposure | 1 |
|  | 4) Demonstration that the outcome of interest was not present at the start of the study | 1 |
| Comparability | 1) Comparability of cohorts on the basis of the design or analysis | 2 |
| Outcome | 1) Assessment of outcome | 1 |
|  | 2) Was follow-up long enough for outcomes to occur | 1 |
|  | 3) Adequacy of follow-up of cohorts | 1 |
| Total Score | 4/4+2/2+3/3 | 9/9 |

**Study: Yahagi, Kazuyuki et al. 2025**

| Selection | 1) Representativeness of the exposed cohort | 1 |
| --- | --- | --- |
|  | 2) Selection of the non-exposed cohort | 1 |
|  | 3) Ascertainment of exposure | 1 |
|  | 4) Demonstration that the outcome of interest was not present at the start of the study | 1 |
| Comparability | 1) Comparability of cohorts on the basis of the design or analysis | 1 |
| Outcome | 1) Assessment of outcome | 1 |
|  | 2) Was follow-up long enough for outcomes to occur | 1 |
|  | 3) Adequacy of follow-up of cohorts | 1 |
| Total Score | 4/4+1/2+3/3 | 8/9 |

**Study: Pan, Y. et al. 2017**

| Selection | 1) Representativeness of the exposed cohort | 0 |
| --- | --- | --- |
|  | 2) Selection of the non-exposed cohort | 1 |
|  | 3) Ascertainment of exposure | 1 |
|  | 4) Demonstration that the outcome of interest was not present at the start of the study | 1 |
| Comparability | 1) Comparability of cohorts on the basis of the design or analysis | 1 |
| Outcome | 1) Assessment of outcome | 1 |
|  | 2) Was follow-up long enough for outcomes to occur | 1 |
|  | 3) Adequacy of follow-up of cohorts | 1 |
| Total Score | 3/4+1/2+3/3 | 7/9 |

# Version 2 of the Cochrane Tool for Assessing Risk of Bias in Randomized Trials (Rob 2) for Randomized Controlled Trials

Note: **Bold** text signifies elevated bias risk, whereas underlined text denotes reduced bias risk.

**Study: Ouweneel, Dagmar M. et al. 2017**

| Domain 1: Risk of bias arising from the randomization process | |
| --- | --- |
| Signalling Questions | Response Options |
| 1.1 Was the allocation sequence random? | Y |
| 1.2 Was the allocation sequence concealed until participants were enrolled and assigned to interventions? | PY |
| 1.3 Did baseline differences between intervention groups suggest a problem with the randomization process? | N |
| Risk-of-bias judgement | Low |
| Optional: What is the predicted direction of bias arising from the randomization  process? | NA |
| Domain 2: Risk of bias due to deviations from intended interventions | |
| Signalling Questions | Response Options |
| 2.1 Were participants aware of their assigned intervention during the trial? | PN |
| 2.2 Were carers and people delivering the interventions aware of participants' assigned intervention during the trial? | **Y** |
| 2.3 If Y/PY/NI to 2.1 or 2.2: Were there deviations from the intended intervention that arose because of the trial context? | PN |
| 2.4 If Y/PY/NI to 2.3: Were these deviations likely to have affected the outcome? | NA |
| 2.5 If Y/PY/NI to 2.4: Were these deviations from intended intervention balanced between groups? | NA |
| 2.6 Was an appropriate analysis used to estimate the effect of assignment to intervention? | Y |
| 2.7 If N/PN/NI to 2.6: Was there potential for a substantial impact (on the result) of the failure to analyze participants in the group to which they were randomized? | NA |
| Risk-of-bias judgement | Some concerns |
| Optional: What is the predicted direction of bias due to deviations from intended  interventions? | NA |
| Domain 3: Missing outcome data | |
| Signalling Questions | Response Options |
| 3.1 Were data for this outcome available for all, or nearly all, participants randomized? | Y |
| 3.2 If N/PN/NI to 3.1: Is there evidence that the result was not biased by missing outcome data? | NA |
| 3.3 If N/PN to 3.2: Could missingness in the outcome depend on its true value? | NA |
| 3.4 If Y/PY/NI to 3.3: Is it likely that missingness in the outcome depended on its true value? | NA |
| Risk-of-bias judgement | Low |
| Optional: What is the predicted direction of bias due to missing outcome data? | NA |
| Domain 4: Risk of bias in measurement of the outcome | |
| Signalling Questions | Response Options |
| 4.1 Was the method of measuring the outcome inappropriate? | N |
| 4.2 Could measurement or ascertainment of the outcome have differed between intervention groups? | N |
| 4.3 If N/PN/NI to 4.1 and 4.2: Were outcome assessors aware of the intervention received by study participants? | PN |
| 4.4 If Y/PY/NI to 4.3: Could assessment of the outcome have been influenced by knowledge of intervention received? | NA |
| 4.5 If Y/PY/NI to 4.4: Is it likely that assessment of the outcome was influenced by knowledge of intervention received? | NA |
| Risk-of-bias judgement | Low |
| Optional: What is the predicted direction of bias in measurement of the outcome? | NA |
| Domain 5: Risk of bias in selection of the reported result | |
| Signalling Questions | Response Options |
| 5.1 Were the data that produced this result analyzed in accordance with a pre-specified analysis plan that was finalized before unblinded outcome data were available for analysis? | Y |
| 5.2 Is the numerical result being assessed likely to have been selected, on the basis of the results, from multiple eligible outcome measurements (e.g. scales, definitions, time points) within the outcome domain? | N |
| 5.3 Is the numerical result being assessed likely to have been selected, on the basis of the results, from multiple eligible analyses of the data? | N |
| Risk-of-bias judgement | Low |
| Optional: What is the predicted direction bias due to selection of the reported results? | NA |
| Overall risk of bias | |
| Risk-of-bias judgement | Low |
| Optional: What is the overall predicted direction of bias for this outcome? | NA |

**Study: Møller, J. E. et al. 2024**

| Domain 1: Risk of bias arising from the randomization process | |
| --- | --- |
| Signalling Questions | Response Options |
| 1.1 Was the allocation sequence random? | Y |
| 1.2 Was the allocation sequence concealed until participants were enrolled and assigned to interventions? | PY |
| 1.3 Did baseline differences between intervention groups suggest a problem with the randomization process? | N |
| Risk-of-bias judgement | Low |
| Optional: What is the predicted direction of bias arising from the randomization  process? | NA |
| Domain 2: Risk of bias due to deviations from intended interventions | |
| Signalling Questions | Response Options |
| 2.1 Were participants aware of their assigned intervention during the trial? | **Y** |
| 2.2 Were carers and people delivering the interventions aware of participants' assigned intervention during the trial? | **Y** |
| 2.3 If Y/PY/NI to 2.1 or 2.2: Were there deviations from the intended intervention that arose because of the trial context? | PN |
| 2.4 If Y/PY/NI to 2.3: Were these deviations likely to have affected the outcome? | NA |
| 2.5 If Y/PY/NI to 2.4: Were these deviations from intended intervention balanced between groups? | NA |
| 2.6 Was an appropriate analysis used to estimate the effect of assignment to intervention? | Y |
| 2.7 If N/PN/NI to 2.6: Was there potential for a substantial impact (on the result) of the failure to analyze participants in the group to which they were randomized? | NA |
| Risk-of-bias judgement | Some concerns |
| Optional: What is the predicted direction of bias due to deviations from intended  interventions? | NA |
| Domain 3: Missing outcome data | |
| Signalling Questions | Response Options |
| 3.1 Were data for this outcome available for all, or nearly all, participants randomized? | Y |
| 3.2 If N/PN/NI to 3.1: Is there evidence that the result was not biased by missing outcome data? | NA |
| 3.3 If N/PN to 3.2: Could missingness in the outcome depend on its true value? | NA |
| 3.4 If Y/PY/NI to 3.3: Is it likely that missingness in the outcome depended on its true value? | NA |
| Risk-of-bias judgement | Low |
| Optional: What is the predicted direction of bias due to missing outcome data? | NA |
| Domain 4: Risk of bias in measurement of the outcome | |
| Signalling Questions | Response Options |
| 4.1 Was the method of measuring the outcome inappropriate? | N |
| 4.2 Could measurement or ascertainment of the outcome have differed between intervention groups? | N |
| 4.3 If N/PN/NI to 4.1 and 4.2: Were outcome assessors aware of the intervention received by study participants? | PN |
| 4.4 If Y/PY/NI to 4.3: Could assessment of the outcome have been influenced by knowledge of intervention received? | NA |
| 4.5 If Y/PY/NI to 4.4: Is it likely that assessment of the outcome was influenced by knowledge of intervention received? | NA |
| Risk-of-bias judgement | Low |
| Optional: What is the predicted direction of bias in measurement of the outcome? | NA |
| Domain 5: Risk of bias in selection of the reported result | |
| Signalling Questions | Response Options |
| 5.1 Were the data that produced this result analyzed in accordance with a pre-specified analysis plan that was finalized before unblinded outcome data were available for analysis? | Y |
| 5.2 Is the numerical result being assessed likely to have been selected, on the basis of the results, from multiple eligible outcome measurements (e.g. scales, definitions, time points) within the outcome domain? | N |
| 5.3 Is the numerical result being assessed likely to have been selected, on the basis of the results, from multiple eligible analyses of the data? | N |
| Risk-of-bias judgement | Low |
| Optional: What is the predicted direction bias due to selection of the reported results? | NA |
| Overall risk of bias | |
| Risk-of-bias judgement | Low |
| Optional: What is the overall predicted direction of bias for this outcome? | NA |

# R Script-Network Meta

library(gemtc)

library(netmeta)

library(metafor)

setwd("D:\\Working file")

data <- read.csv("", sep = ",", header = T)

network <- mtc.network(data)

summary(network)

print(network)

plot(network)

sample_data <- aggregate(sampleSize ~ treatment,

data = network$data.ab,

FUN = sum,

na.rm = TRUE)

node_sizes <- setNames(sqrt(sample_data$sampleSize) * 2,

sample_data$treatment)

node_sizes <- node_sizes[network$treatments$id]

tiff("network_plot.tiff", width = 8, height = 6, units = "in", res = 300, compression = "lzw")

plot(network, use.description = TRUE,

vertex.label = NA,

vertex.size =node_sizes / 8,

vertex.shape = "circle",

vertex.label.cex = 1.5,

vertex.label.color = "blank",

vertex.label.dist = 12,

vertex.label.degree = -pi/40,

vertex.color = "steelblue",

dynamic.edge.width = TRUE,

edge.color = "black"

)

dev.off()

plot(network)

model <- mtc.model(network, type = "consistency",

n.chain = 4,

likelihood = "binom",

link = "logit",

linearModel = "random"

)

results <- mtc.run(model, n.adapt = 5000,

n.iter = 20000,

thin = 1)

summary(results)

gelman.diag(results)

tiff("gelman_plot.tiff", width = 8, height = 6, units = "in", res = 300, compression = "lzw")

gelman.plot(results)

dev.off()

tiff("forest_control.tiff", width = 8, height = 6, units = "in", res = 300, compression = "lzw")

gemtc::forest(relative.effect(results, "Control"))

dev.off()

ranks <- rank.probability(results, preferredDirection = -1)

print(ranks)

sucra <- function(ranks) {

apply(ranks, 1, function(p) {

a <- length(p)

sum(cumsum(p[-a]))/(a-1)

})

}

a <- sucra(ranks)

a <- round(exp(relative.effect.table(results)),2)

write.csv(a, "leaguetable.csv")

modelume <- mtc.model(network,

n.chain = 4,

likelihood = "binom",

link = "logit",

linearModel = "random")

results <- mtc.run(modelume, n.adapt = 5000,

n.iter = 20000,

thin = 1)

summary(results)

resultnodesplit <- mtc.nodesplit(network,

n.adapt = 20000,

n.iter = 50000,

thin = 1,

n.chain = 4,

likelihood = "binom",

linearModel = "random")

b <- summary(resultnodesplit)

tiff("resultnodesplit.tiff", width = 8, height = 20, units = "in", res = 300, compression = "lzw")

plot(b)

dev.off()

c <- ranks[,]

library(reshape2)

d <- melt(c)

e <- as.data.frame(d)

library(ggplot2)

ranksplotemm <- ggplot(e, aes(x = Var2, y = value, colour = Var1))+geom_line(size = 1)

print(ranksplotemm)

ranksplot <- ranksplotemm+

geom_point(size = 2)+

theme(axis.text = element_text(size = 12),

axis.title = element_text(size = 15),

panel.background = element_blank(),

axis.line = element_line(color = "black"),

legend.title = element_blank(),

legend.position = "bottom")+

scale_y_continuous(

name = "Probability",

breaks = seq(0, 1, by = 0.2),

limits = c(0, 1))+

scale_x_continuous(

name = "Rank",

breaks = seq(1, 7, by = 1),

limits = c(1, 7))+

coord_fixed(ratio = 5/1)

tiff("ranksplot.tiff", width = 8, height = 20, units = "in", res = 300, compression = "lzw")

print(ranksplot)

dev.off()

f <- t(apply(ranks, 1, cumsum))

g <- melt(f)

h <- as.data.frame(g)

cumprobability1 <- ggplot(h, aes(x = Var2, y = value,

colour = Var1))+

geom_line(size = 1)

cumprobability <- cumprobability1+

geom_point(size = 2)+

theme(axis.text = element_text(size = 12),

axis.title = element_text(size = 15),

panel.background = element_blank(),

axis.line = element_line(color = "black"),

legend.title = element_blank(),

legend.position = "bottom")+

scale_y_continuous(

name = "Probability",

breaks = seq(0, 1, by = 0.2),

limits = c(0, 1))+

scale_x_continuous(

name = "Rank",

breaks = seq(1, 7, by = 1),

limits = c(1, 7))+

coord_fixed(ratio = 5/1)

tiff("cumprobability.tiff", width = 8, height = 20, units = "in", res = 300, compression = "lzw")

print(cumprobability)

dev.off()

# R Script-Pairwise Meta

setwd("D:\\Working file")

mydata <- read.csv("")

library(meta)

library(metafor)

mydata

names(mydata)

author <- mydata$Author

year <- mydata$Year

event1 <- mydata$event1

total1 <- mydata$total1

event2 <- mydata$event2

total2 <- mydata$total2

metadata <- metabin(

event.e = event1,

n.e = total1,

event.c = event2,

n.c = total2,

sm = "OR",

common = FALSE,

random = TRUE,

studlab = paste(author, year, sep = "\t"),

)

print(metadata)

tiff("forest plot.tiff", width = 16, height = 8, units = "in", res = 300, compression = "lzw")

forest(metadata)

dev.off()

tiff("labbe plot.tiff", width = 16, height = 8, units = "in", res = 300, compression = "lzw")

labbe(metadata)

dev.off()

tiff("funnel plot.tiff", width = 16, height = 8, units = "in", res = 300, compression = "lzw")

funnel(metadata)

dev.off()

tiff("sensitivity analysis plot.tiff", width = 16, height = 8, units = "in", res = 300, compression = "lzw")

forest(metainf(metadata, pooled = "Random"))

dev.off()

tf0 <- trimfill(metadata)

tiff("trimfill plot.tiff", width = 16, height = 8, units = "in", res = 300, compression = "lzw")

funnel(tf0)

dev.off()

TE <- metadata$TE

seTE <- metadata$seTE

metafor_model <- rma(yi = TE, sei = seTE, method = "REML")

egger_metafor <- regtest(metafor_model)

print(egger_metafor)

Convergence in the Bayesian network meta-analysis was evaluated via Gelman-Rubin diagnostics, with potential scale reduction factors (R̂) below 1.05 for all parameters, confirming satisfactory convergence.

# Gelman-Rubin Diagnostic Plot for Convergence Assessment

**(R̂: Median and 97.5% Quantile)**

**Short-Term Mortality**

**Bleeding**

**Stroke**

# DIC Values for the Inconsistency and Consistency Models

Inconsistency model

-- Model fit (residual deviance):

| Dbar | pD | DIC |
| --- | --- | --- |
| 46.03685 | 43.93938 | 89.97623 |

I^2 = 0.08%

Consistency model

-- Model fit (residual deviance):

| Dbar | pD | DIC |
| --- | --- | --- |
| 45.88505 | 43.80850 | 89.69355 |

I^2 = 0%

# Results of Node-splitting

# Forest Plot, Funnel Plot, Sensitivity Analysis Plot, Labbe Plot, and Trimfill Plot

**Short-term mortality, IABP vs. Control (Observational evidence)**

**A. Forest plot**

**B.** **Funnel plot**

**C. Sensitivity analysis plot**

**D. Labbe plot**

**E. Trimfill plot**

**Short-term mortality, IABP vs. Control (Propensity-matched observational evidence)**

**A. Forest plot**

**B.** **Funnel plot**

**Not applicable**

**C. Sensitivity analysis plot**

**Not applicable**

**D. Labbe plot**

**Not applicable**

**E. Trimfill plot**

**Not applicable**

**Short-term mortality, ECMO vs. Control (Observational evidence)**

**A. Forest plot**

**B.** **Funnel plot**

**C. Sensitivity analysis plot**

**D. Labbe plot**

**E. Trimfill plot**

**Short-term mortality, ECMO vs. Control after removing heterogeneity (Observational evidence)**

**A. Forest plot**

**B.** **Funnel plot**

**C. Sensitivity analysis plot**

**D. Labbe plot**

**E. Trimfill plot**

**Short-term mortality, Impella vs. Control (Observational and RCT evidence)**

**A. Forest plot**

**B.** **Funnel plot**

**Not applicable**

**C. Sensitivity analysis plot**

**Not applicable**

**D. Labbe plot**

**Not applicable**

**E. Trimfill plot**

**Not applicable**

**Short-term mortality, Impella vs. IABP (Observational and RCT evidence)**

**A. Forest plot**

**B.** **Funnel plot**

**C. Sensitivity analysis plot**

**D. Labbe plot**

**E. Trimfill plot**

**Short-term mortality, Impella vs. IABP after removing heterogeneity (Observational and RCT evidence)**

**A. Forest plot**

**B.** **Funnel plot**

**Not applicable**

**C. Sensitivity analysis plot**

**Not applicable**

**D. Labbe plot**

**Not applicable**

**E. Trimfill plot**

**Not applicable**

**Short-term mortality, Impella vs. IABP (Observational evidence)**

**A. Forest plot**

**B.** **Funnel plot**

**Not applicable**

**C. Sensitivity analysis plot**

**Not applicable**

**D. Labbe plot**

**Not applicable**

**E. Trimfill plot**

**Not applicable**

**Short-term mortality, IABP vs. ECMO (Observational evidence)**

**A. Forest plot**

**B.** **Funnel plot**

**C. Sensitivity analysis plot**

**D. Labbe plot**

**E. Trimfill plot**

**Bleeding, IABP vs. Control (Observational evidence)**

**A. Forest plot**

**B.** **Funnel plot**

**C. Sensitivity analysis plot**

**D. Labbe plot**

**E. Trimfill plot**

**Bleeding, Impella vs. IABP (Observational and RCT evidence)**

**A. Forest plot**

**B.** **Funnel plot**

**Not applicable**

**C. Sensitivity analysis plot**

**Not applicable**

**D. Labbe plot**

**Not applicable**

**E. Trimfill plot**

**Not applicable**

**Stroke, IABP vs. Control (Observational evidence)**

**A. Forest plot**

**B.** **Funnel plot**

**C. Sensitivity analysis plot**

**D. Labbe plot**

**E. Trimfill plot**

**Stroke, Impella vs. IABP (Observational and RCT evidence)**

**A. Forest plot**

**B.** **Funnel plot**

**C. Sensitivity analysis plot**

**D. Labbe plot**

**E. Trimfill plot**

**Stroke, Impella vs. IABP after removing heterogeneity (Observational and RCT evidence)**

**A. Forest plot**

**B.** **Funnel plot**

**Not applicable**

**C. Sensitivity analysis plot**

**Not applicable**

**D. Labbe plot**

**Not applicable**

**E. Trimfill plot**

**Not applicable**

**Stroke, Impella vs. IABP (Observational evidence)**

**A. Forest plot**

**B.** **Funnel plot**

**Not applicable**

**C. Sensitivity analysis plot**

**Not applicable**

**D. Labbe plot**

**Not applicable**

**E. Trimfill plot**

**Not applicable**

**Stroke, Impella vs. Control (Observational and RCT evidence)**

**A. Forest plot**

**B.** **Funnel plot**

**Not applicable**

**C. Sensitivity analysis plot**

**Not applicable**

**D. Labbe plot**

**Not applicable**

**E. Trimfill plot**

**Not applicable**

**1-year mortality, IABP vs. Control (Observational evidence)**

**A. Forest plot**

**B.** **Funnel plot**

**C. Sensitivity analysis plot**

**D. Labbe plot**

**E. Trimfill plot**

**1-year mortality, IABP vs. Control after removing heterogeneity (Observational evidence)**

**A. Forest plot**

**B. Funnel plot**

**Not applicable**

**C. Sensitivity analysis plot**

**Not applicable**

**D. Labbe plot**

**Not applicable**

**E. Trimfill plot**

**Not applicable**

**Acute kidney injury, Impella vs. IABP (Observational evidence)**

**A. Forest plot**

**B.** **Funnel plot**

**Not applicable**

**C. Sensitivity analysis plot**

**Not applicable**

**D. Labbe plot**

**Not applicable**

**E. Trimfill plot**

**Not applicable**

**Acute kidney injury leading to hemodialysis, Impella vs. IABP (Observational evidence)**

**A. Forest plot**

**B.** **Funnel plot**

**Not applicable**

**C. Sensitivity analysis plot**

**Not applicable**

**D. Labbe plot**

**Not applicable**

**E. Trimfill plot**

**Not applicable**

# SUCRA Rank Plot (Sensitivity Analysis: rhBNP Excluded)

# League Table of Treatment Comparisons (Sensitivity Analysis: rhBNP Excluded)

| Control |  |  | Control | 1.16 (0.38, 3.59) | 2.78 (0.85, 8.1) |
| --- | --- | --- | --- | --- | --- |
| 0.76 (0.36, 1.61) | ECMO |  | 0.67 (0.27, 1.39) | IABP | 2.4 (0.82, 6.25) |
| **0.17 (0.03, 0.97)** | 0.22 (0.03, 1.38) | ECMO + Impella | **0.39 (0.12, 0.84)** | 0.6 (0.21, 1.2) | Impella |
| 0.96 (0.61, 1.49) | 1.26 (0.62, 2.57) | **5.81 (1, 33.53)** | IABP |  |  |
| 4.01 (0.6, 28.27) | 5.29 (0.72, 40.94) | **24.36 (1.88, 318.56)** | 4.2 (0.66, 28.36) | IABP + ECMO |  |
| 0.65 (0.29, 1.48) | 0.85 (0.32, 2.31) | 3.92 (0.82, 18.81) | 0.67 (0.31, 1.5) | 0.16 (0.02, 1.21) | Impella |
| Odds ratios (ORs) with 95% credible intervals are calculated. Values > 1.00 indicate higher odds of the outcome in the column group compared to the row group (column vs. row). | | | | | |
|  | | | | | |

# References of Included Studies

Randomized Controlled Trials (RCTs):

1.	Møller JE, Engstrøm T, Jensen LO, et al. Microaxial flow pump or standard care in infarct-related cardiogenic shock. New England Journal of Medicine 2024;390:1382‐1393.

2.	Ouweneel DM, Eriksen E, Sjauw KD, et al. Percutaneous mechanical circulatory support versus intra-aortic balloon pump in cardiogenic shock after acute myocardial infarction. Journal of the American College of Cardiology 2017;69:278-287.

Cohort Studies:

3.	Yahagi K, Gonda Y, Yoshiura D, et al. Impact of lactate levels on admission in STEMI patients with cardiogenic shock treated with IMPELLA. Heart and Vessels 2025.

4.	Diaz-Arocutipa C, Bueno H, Moreno G, et al. Differences on in-hospital outcomes in patients with cardiogenic shock due to STEMI versus NSTEMI using a nationwide database. Shock 2025;63:36-42.

5.	Yang H, Luo L, Song Y, et al. ECMO versus IABP for patients with STEMI complicated by cardiogenic shock undergoing primary PCI: A Chinese national study and propensity-matched analysis. Hellenic Journal of Cardiology 2024.

6.	Somaschini A, Cornara S, Leonardi S, et al. Beneficial effects of IABP in anterior myocardial infarction complicated by cardiogenic shock. Medicina 2023;59.

7.	Briasoulis A, Kampaktsis P, Emfietzoglou M, et al. Temporary mechanical circulatory support in cardiogenic shock due to ST-elevation myocardial infarction: Analysis of the national readmissions database. Angiology 2023;74:31-38.

8.	Thakkar S, Patel HP, Kumar A, et al. Outcomes of Impella compared with intra-aortic balloon pump in ST-elevation myocardial infarction complicated by cardiogenic shock. American Heart Journal Plus: Cardiology Research and Practice 2021;12:100067-100067.

9.	Pahuja M, Chehab O, Ranka S, et al. Incidence and clinical outcomes of stroke in ST-elevation myocardial infarction and cardiogenic shock. Catheterization and Cardiovascular Interventions: Official Journal of the Society for Cardiac Angiography & Interventions 2021;97:217-225.

10.	Lan WR, Lin SI, Liao FC, et al. The impact of intra-aortic balloon pump in elderly with ST-segment elevation myocardial infarction undergoing primary percutaneous cardiovascular intervention: Benefits and risks? International Journal of Gerontology 2021;15:195-200.

11.	Singh SSA, Das De S, Nappi F, et al. Mechanical circulatory support for refractory cardiogenic shock post-acute myocardial infarction - a decade of lessons. Journal of Thoracic Disease 2019;11:542-548.

12.	Petrovic M, Jarakovic M, Cankovic M, et al. Complete percutaneous myocardial revascularization in patients with STEMI complicated by cardiogenic shock. Vojnosanitetski Pregled 2019;76:152-160.

13.	Boudiche S, Guermazi O, El Ayech F, et al. Outcome predictors of cardiogenic shock complicating ST-segment elevation myocardial infarction. La Tunisie Medicale 2019;97:476-483.

14.	Pan Y, Lu Z, Hang J, et al. Effects of low-dose recombinant human brain natriuretic peptide on anterior myocardial infarction complicated by cardiogenic shock. Brazilian Journal of Cardiovascular Surgery 2017;32:96-103.

15.	de la Espriella-Juan R, Valls-Serral A, Trejo-Velasco B, et al. Impact of intra-aortic balloon pump on short-term clinical outcomes in ST-elevation myocardial infarction complicated by cardiogenic shock: A "real life" single center experience. Medicina Intensiva 2017;41:86-93.

16.	Iqbal MB, Robinson SD, Ding L, et al. Intra-aortic balloon pump counterpulsation during primary percutaneous coronary intervention for ST-elevation myocardial infarction and cardiogenic shock: Insights from the British Columbia Cardiac Registry. Plos One 2016;11:e0148931.

17.	Jensen JK, Thayssen P, Antonsen L, et al. Influence of cardiogenic shock with or without the use of intra-aortic balloon pump on mortality in patients with ST-segment elevation myocardial infarction. International Journal of Cardiology Heart & Vasculature 2015;6:19-24.

18.	Pathak R, Aryal MR, Karmacharya P, et al. Mortality in intra-aortic balloon pump therapy in patients with ST elevation myocardial infarction and cardiogenic shock: Data from nationwide inpatient sample. International Journal of Cardiology 2014;176:279-80.

19.	Dziewierz A, Siudak Z, Rakowski T, et al. Impact of intra-aortic balloon pump on long-term mortality of unselected patients with ST-segment elevation myocardial infarction complicated by cardiogenic shock. Postepy W Kardiologii Interwencyjnej 2014;10:175-180.

20.	Sjauw KD, Engstrom AE, Vis MM, et al. Efficacy and timing of intra-aortic counterpulsation in patients with ST-elevation myocardial infarction complicated by cardiogenic shock. Netherlands Heart Journal 2012;20:402-409.

21.	Sheu J-J, Tsai T-H, Lee F-Y, et al. Early extracorporeal membrane oxygenator-assisted primary percutaneous coronary intervention improved 30-day clinical outcomes in patients with ST-segment elevation myocardial infarction complicated with profound cardiogenic shock. Critical Care Medicine 2010;38:1810-1817.

22.	Gu J, Hu W, Xiao H, et al. Intra-aortic balloon pump improves clinical prognosis and attenuates C-reactive protein level in acute STEMI complicated by cardiogenic shock. Cardiology 2010;117:75-80.

Updated Search Through September 2026:

1. Sørensen R, Møller JE, Vandenbriele C, et al. Bleeding in patients with infarct-related cardiogenic shock: a DanGer Shock substudy. JACC Cardiovascular Interventions 2026;19:302-312.
